# An Abscisic Acid-Independent Oxylipin Pathway Controls Stomatal Closure and Immune Defense in *Arabidopsis*

**DOI:** 10.1371/journal.pbio.1001513

**Published:** 2013-03-19

**Authors:** Jean-Luc Montillet, Nathalie Leonhardt, Samuel Mondy, Sylvain Tranchimand, Dominique Rumeau, Marie Boudsocq, Ana Victoria Garcia, Thierry Douki, Jean Bigeard, Christiane Laurière, Anne Chevalier, Carmen Castresana, Heribert Hirt

**Affiliations:** 1 CEA Cadarache, Direction des Sciences du Vivant (DSV), Institut de Biologie Environnementale et Biotechnologie (IBEB), Service de Biologie Végétale et de Microbiologie Environnementale (SBVME), Laboratoire d'Ecophysiologie Moléculaire des Plantes, Unité Mixte de Recherche (UMR) 7265, Centre National de la Recherche Scientifique (CNRS)/Commissariat à l'Energie Atomique (CEA)/Aix-Marseille Université, Saint-Paul-lez-Durance, France; 2 CEA Cadarache, DSV, IBEB, SBVME, Laboratoire de Biologie du Développement des Plantes, UMR 7265, CNRS/CEA/Aix-Marseille Université, Saint-Paul-lez-Durance, France; 3 CEA Cadarache, DSV, IBEB, SBVME, Laboratoire des Echanges Membranaires et Signalisation, UMR 7265, CNRS/CEA/Aix-Marseille Université, Saint-Paul-lez-Durance, France; 4 CNRS, Institut des Sciences du Végétal, Unité Propre de Recherche 2355, Gif-sur-Yvette Cedex, France; 5 Unité de Recherche en Génomique Végétale Plant Genomics, Institut National de la Recherche Agronomique/CNRS/University of Evry, Evry, France; 6 Laboratoire “Lésions des Acides Nucléiques”, Service de Chimie Inorganique et Biologique, UMR-E3 CEA/Université Joseph Fourier–Grenoble 1, Institut Nanosciences et Cryogénie, Grenoble, France; 7 Centro Nacional de Biotecnología, Consejo Superior de Investigaciones Cientificas, Campus Universidad Autónoma, Cantoblanco, Madrid, Spain; The University of North Carolina at Chapel Hill, United States of America

## Abstract

Plant stomata function in innate immunity against bacterial invasion and abscisic acid (ABA) has been suggested to regulate this process. Using genetic, biochemical, and pharmacological approaches, we demonstrate that (i) the *Arabidopsis thaliana* nine-specific-lipoxygenase encoding gene, *LOX1*, which is expressed in guard cells, is required to trigger stomatal closure in response to both bacteria and the pathogen-associated molecular pattern flagellin peptide flg22; (ii) LOX1 participates in stomatal defense; (iii) polyunsaturated fatty acids, the LOX substrates, trigger stomatal closure; (iv) the LOX products, fatty acid hydroperoxides, or reactive electrophile oxylipins induce stomatal closure; and (v) the flg22-mediated stomatal closure is conveyed by both LOX1 and the mitogen-activated protein kinases MPK3 and MPK6 and involves salicylic acid whereas the ABA-induced process depends on the protein kinases OST1, MPK9, or MPK12. Finally, we show that the oxylipin and the ABA pathways converge at the level of the anion channel SLAC1 to regulate stomatal closure. Collectively, our results demonstrate that early biotic signaling in guard cells is an ABA-independent process revealing a novel function of LOX1-dependent stomatal pathway in plant immunity.

## Abstract 2

In *Arabidopsis* the stomatal defense response, a feature of the innate immunity in plants, involves oxylipin-mediated mechanisms that are independent of the phytohormone abscisic acid.

## Author Summary

Stomata are microscopic pores that are present in the epidermis of the aerial parts of higher plants, such as the leaves. These pores, which are flanked by a pair of cells called guard cells, regulate transpiration and the exchange of gas between leaves and the atmosphere. It is well documented that the phytohormone abscisic acid (ABA) is a key regulator that controls the osmotic pressure in guard cells, allowing pore size to be adjusted in response to environmental conditions. Recently, stomata have also been shown to play an important role in the innate immune response. Indeed, upon contact with microbes, plants actively close stomata to prevent the entry of microbes and the consequent colonization of host tissue. This response is known as the stomatal defense response. However, the molecular mechanisms that regulate this defense response are not well understood. Using a variety of approaches, we show in this study that *LOX1*, a gene that encodes lipoxygenase (LOX) in guard cells, plays a major role in stomatal defense in the model plant *Arabidopsis thaliana*. Mutations in *LOX1* impair stomatal closure and make plants more susceptible to the bacterium *Pseudomonas syringae pv. tomato*. We also show that several LOX-derived metabolites, the oxylipins, are potent inducers of stomatal closure. Finally, we provide evidence to show that ABA plays only a minor role in stomatal defense response, specifically by modulating this response.

## Introduction

Due to their sessile nature and the lack of an adaptive immune system, plants have evolved processes to synthesize a vast array of secondary metabolites devoted to their protection against pathogens. Some of these compounds, known as oxylipins, originate from the incorporation of one or several oxygen atoms in the carbon chain of polyunsaturated fatty acids (PUFAs), mainly linoleic acid (18∶2), linolenic acid (18∶3), and roughanic acid (16∶3). This first step leading to the formation of oxygenated fatty acids can be initiated either by reactive oxygen species (ROS) or enzymes such as lipoxygenases (LOXs), α-dioxygenases, or cytochromes P450. The primary hydroperoxy or hydroxy fatty acids can subsequently be converted into a plethora of metabolites, including aldehydes and oxo-acids, epoxydes, epoxy alcohols, hydroxides, divinyl ethers, and cyclized compounds such as jasmonates or phytoprostanes [1]–[4], which are involved in different aspects of plant physiology such as development, fertility, senescence, programmed cell death, and defense [3],[5]–[7],[8]–[10].

The 9- and 13-specific LOXs that introduce two atoms of oxygen on carbon 9 or 13 of octadecanoic fatty acids, respectively, contribute to the synthesis of a great number of oxylipins. LOX genes are up-regulated and LOX-derived oxylipins are synthesized upon stress and pathogen challenges, suggesting that LOXs play a role in stress adaptation and pathogen defense [11],[12]. The 13-LOX product JA has received much attention as it has been demonstrated to be involved in almost all above-mentioned physiological processes [10],[13]–[15]. The JA signaling pathway is now well documented involving the conjugate jasmonate-Isoleucine (JA-Ile) as the active form of jasmonic acid (JA), the F-box protein Coronatine Insensitive 1 (COI1) as receptor, and Jasmonate Zim Domain (JAZ) transcription factors as repressors whose degradation through the 26S proteasome pathway leads to the transcription of JA-responsive genes [16]. The 9-LOX-derived products have also been identified as signaling molecules regulating plant defense, cell death, as well as lateral root development and singlet oxygen-mediated stress [7],[17]–[21]. In contrast to JA, the mechanisms whereby 9-LOX products exert their biological functions are still unknown. Due to their enzymatic activity LOX can produce reactive electrophile species oxylipin (RES oxylipin) from fatty acid hydroperoxides. Recently, the mechanisms underlying RES oxylipin signaling has been uncovered. RES oxylipins possess reactive α,β-unsaturated carbonyl or epoxide groups and can add to thiol- or amine-containing compounds, such as glutathione (GSH), proteins, or nucleic acids [22]–[27]. The chemical reactivity of RES oxylipins toward the antioxidant GSH can modify the cellular redox state. RES oxylipins can also target transcription factors and the TGA transcription factors (TGA2, TGA5, and TGA6) were demonstrated to regulate the RES oxylipin-mediated gene transcription [26].

The development of transcriptomic studies and the possibility to access analyses of cell-type-specific gene expressions have enabled us to refine our understanding on the role of genes in plant-specific organs or cells. In this respect, the analyses of cell-type-specific leaf transcriptome [28] allowed much progress in the knowledge of guard cell signaling pathways that contribute to stomatal movements in response to various environmental stresses [29]–[31].

Recently, a role of guard cells as an active plant defense mechanism against pathogens has been highlighted by the finding that stomata also function in innate immunity against bacterial invasion [32]–[34]. Soon after pathogen recognition, the two guard cells that flank the stomata actively restrict the opening size preventing entry of microbes and host tissue colonization. This response is referred to as the stomatal defense [35], and it has been suggested that the abscisic acid (ABA) was a key regulator of the pathogen-mediated stomatal closure [32].

The analysis of the cell-type-specific leaf transcriptome of Arabidopsis [28] allowed us to identify the guard cell specifically expressed *LOX* encoding genes *LOX1* and *LOX6*. By multiple approaches we demonstrate that, in contrast to *LOX6*, *LOX1* plays a major role in the control of stomatal defense and plant innate immunity. We provide evidence that the MAPKs MPK3 and MPK6 and salicylate participate in a LOX1-specific stomatal pathway to respond to pathogens and PAMPs. Moreover, we show that the ABA pathway including the protein kinases OST1, MPK9, and MPK12 only contribute to a small extent to the stomatal response to pathogens. Overall, our data reveal the functioning of an oxylipin- and an ABA-dependent pathway that converge at the level of the anion channel SLAC1 to regulate stomatal closure.

## Results

### The 9-Specific LOX Encoding Gene *LOX1* Is Expressed in Guard Cells

Cell-type-specific transcriptome analysis of Arabidopsis leaves [28] revealed striking differences in the expression of *LOX* genes (Figure S1A). Genes encoding 13-LOXs, *LOX2*, *LOX3*, and *LOX4* [36] were expressed in mesophyll cells, while *LOX6* expression was restricted to guard cells. Conversely, expression of the 9-LOX encoding gene *LOX1* was detected in guard cells but not in mesophyll cells. Microarray analyses were confirmed by RT-PCR for *LOX1*, expressed in guard cells and *LOX2*, *LOX3*, and *LOX4* whose expression is restricted to mesophyll cells (Figure S1B).

### *Lox1* Mutants Are More Susceptible to the Bacterial Pathogen *Pseudomonas syringae pv. tomato* (*Pst*) DC3000

**Figure 1:**
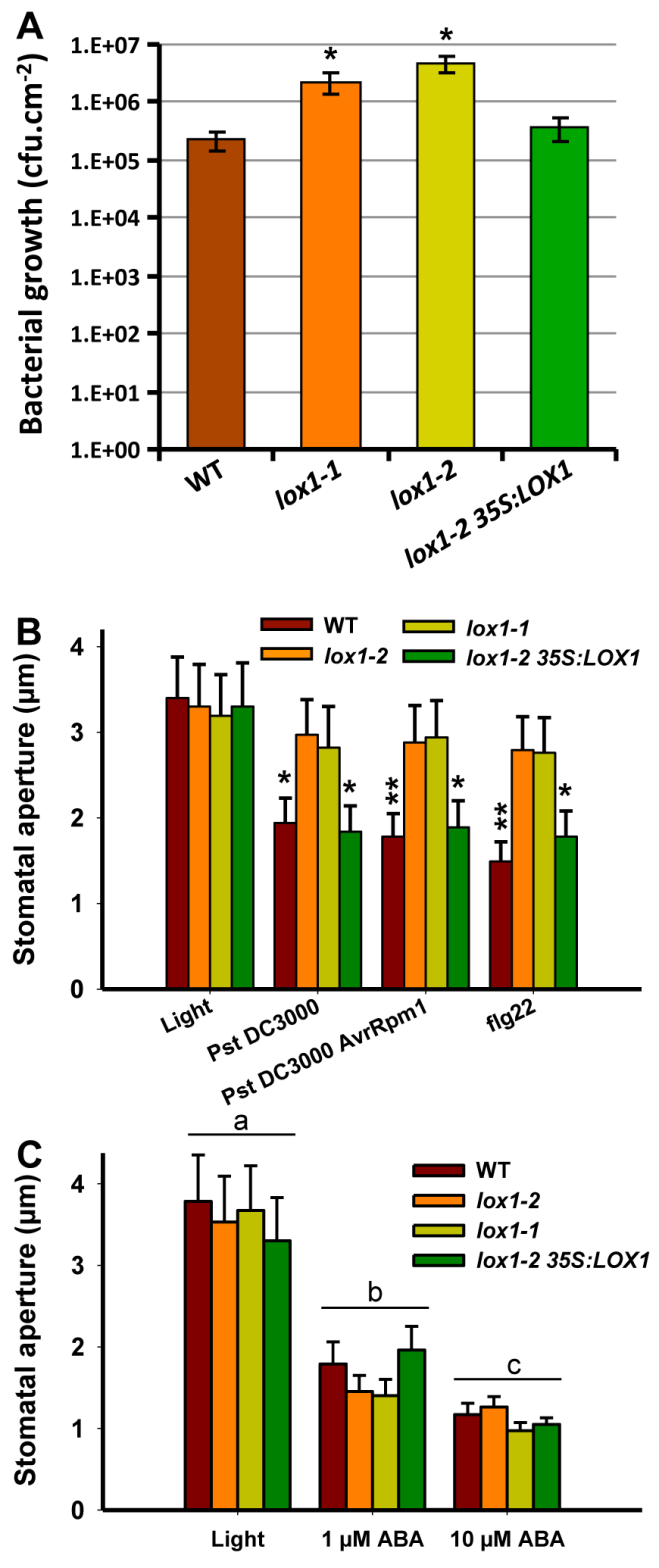
Stomatal defense and stomatal closure responses of *lox1* mutant lines. (A) Growth of *Pst* DC3000 was assessed 3 days after spray inoculation at 5×10^7^ cfu/mL. Bars represent means of five replicates ± SE. Similar results were obtained in three independent experiments. (B) Stomatal apertures were measured on 2 h light-preincubated epidermal peels, incubated for 1 h with bacterial suspensions (10^8^ cfu/mL) or 2.5 h with flg22 (5 µM) or (C) with ABA. Data represent means ± SD of three independent experiments, and values marked with asterisks were statistically different from those of the corresponding light controls. In (C), means marked with identical letters were not statistically different (a: *F* = 0.421, *df* = 11, *p* = 0.743; b: *F* = 3.683, *df* = 11, *p* = 0.062; c: *F* = 3.726, *df* = 11, *p* = 0.061).

In order to determine whether *LOX1* contributes to stomatal defense, two *lox1* knockout lines (*lox1-1* and *lox1-2*) along with a complemented *lox1-2* (*lox1-2 35S:LOX1*) line and Col-0 as the wild-type (WT) control line were assessed for their resistance to virulent *Pst* DC3000 upon spray inoculations (Figure 1A). The two *lox1* mutant lines displayed more than 10-fold enhanced growth of *Pst* DC3000 as compared to the complemented and WT lines, suggesting that *LOX1* participates in the control of bacterial leaf colonization.

### *Lox1* Mutants Are Compromised in Pathogen and PAMP-Induced Stomatal Closure

To test whether *LOX1* exerts its effect on plant immunity at the guard cell level, we compared the behavior of stomata of *lox1* mutants with WT plants in response to *Pst*. In contrast to WT and *lox1-2 35S:LOX1*, *lox1* mutants were significantly compromised in their ability to close stomata in response to virulent *Pst* DC3000 and avirulent *Pst* DC3000 *AvrRpm1* strains (Figure 1B). Similarly, the flg22-induced stomatal closure was strongly impaired in both *lox1* mutants (Figure 1B). On the contrary, the *lox6-1* mutant line (Methods S1), was not compromised in its ability to close stomata in response to flg22 (Figure S2).

Considering that ABA has been identified as a positive regulator of pathogen and PAMP-induced stomatal closure [33], we investigated also whether *lox1* mutants showed altered sensitivity to ABA. We observed that both *lox1* mutant lines were as sensitive to exogenously applied ABA as WT and *lox1-2 35S:LOX1* (Figure 1C). Together, these results suggest that LOX1 activity mediates pathogen- and flg22-induced stomatal closure independently of ABA.

### PUFAs Induce Stomatal Closure in Col-0 Plants But Fail to Activate This Process in *lox1* Mutants

**Figure 2:**
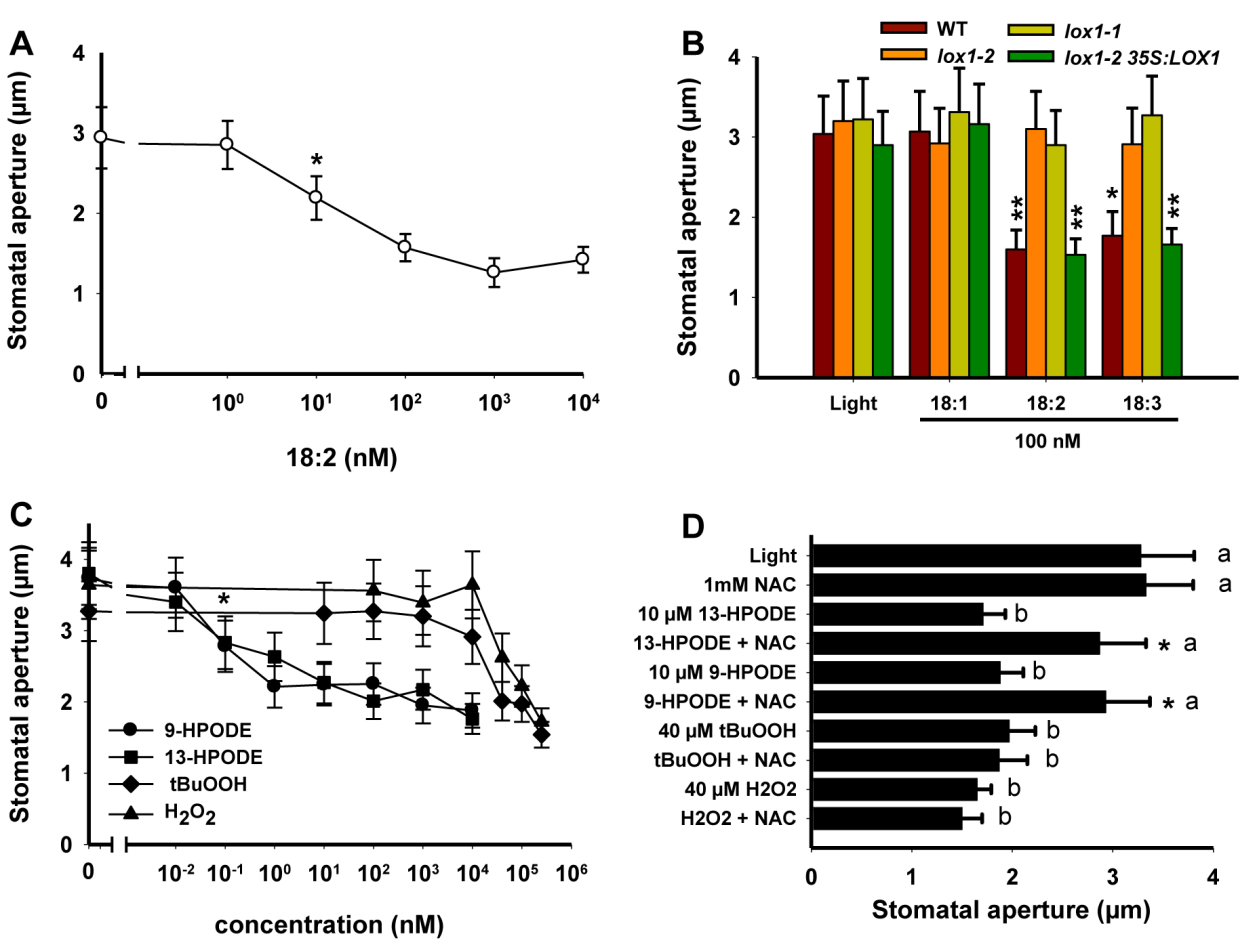
Stomatal closure responses to fatty acids and hydroperoxides. Measurements were performed on 2 h light-preincubated epidermal peels from 4–5-wk-old Arabidopsis plants after 2.5 h incubation, and data represent means ± SD of three independent experiments. (A) Dose response effects of linoleic acid (18∶2) on Col-0 plants. Student's *t* test analysis of means indicates that the effect of 18∶2 becomes significant at 10 nM. (B) Effects of oleic (18∶1), linoleic (18∶2), and linolenic (18∶3) acids at a concentration of 100 nM. Values marked with asterisks were statistically different from the corresponding light controls. (C) Dose response effects of FAHs (9- and 13-HPODE) H_2_O_2_ and t-BuOOH on Col-0 plants. Student's *t* test analysis of means indicates that the effects of 9- and 13-HPODE become significant at 0.1 nM. (D) NAC (1 mM) was added to epidermal peels 30 min prior to addition of hydroperoxides. Values marked with asterisks were significantly different from those of the corresponding sample without NAC pre-incubation, and means marked with identical letters were not statistically different (a: *F* = 0.737, *df* = 11, *p* = 0.559; b: *F* = 1.792, *df* = 11, *p* = 0.189).

Since PUFAs are known LOX substrates, we first tested linoleic acid (18∶2) for its ability to induce stomatal closure. We observed that linoleic acid induced significant stomatal closure at 10 nM (Figure 2A). At 100 nM both linoleic (18∶2) and linolenic acid (18∶3) induced stomatal closure in WT and *lox1-2 35S:LOX1* plants but not in both *lox1* mutants (Figure 2B). In contrast, oleic acid (18∶1), which is not a substrate of LOXs, was unable to trigger stomatal closure in any plant line (Figure 2B). These results strongly suggest that products of LOX activity are able to promote stomatal closure.

### Fatty Acid Hydroperoxides (FAHs) Activate Stomatal Closure at Lower Doses Than H_2_O_2_ or *tert*-Butyl Hydroperoxide (tBuOOH)

LOXs directly generate FAHs by incorporating molecular oxygen into the backbone of PUFAs (Figure S3). We therefore tested the ability of 9- and 13-HPODE to trigger stomatal closure and compared their activity to H_2_O_2_, which was previously shown to mediate ABA-induced stomatal closure [37] and to tBuOOH as a model organic hydroperoxide. We found that 9- and 13-HPODE promoted stomatal closure at considerably lower concentrations than H_2_O_2_ or tBuOOH (Figure 2C), demonstrating that LOX-derived FAHs are potent inducers of stomatal closure.

Oxidative processes arising from increased cellular hydroperoxide concentrations can be effectively prevented by the addition of free radical scavengers including thiol-containing compounds. Among them, N-acetyl cysteine (NAC) is one of the less potent free radical scavengers [38]. Alternatively, thiols can act as nucleophiles to conjugate with reactive electrophiles through the Michael addition [25],[27],[39]. In order to get further insights into the mechanisms at the origin of the biological activities of hydroperoxides, epidermal peels were pre-incubated with 1 mM NAC before treatment with 10 µM 9- or 13-HPODE, 40 µM H_2_O_2_, or tBuOOH (Figure 2D). The inability of NAC to prevent H_2_O_2_- or tBuOOH-induced stomatal closure suggested that NAC did not function as an antioxidant. On the other hand, its specific inhibitory effect on FAHs rather suggested that it could act as a nucleophilic compound.

### Thiol Reagents Are Powerful Activators of Stomatal Closure

**Figure 3:**
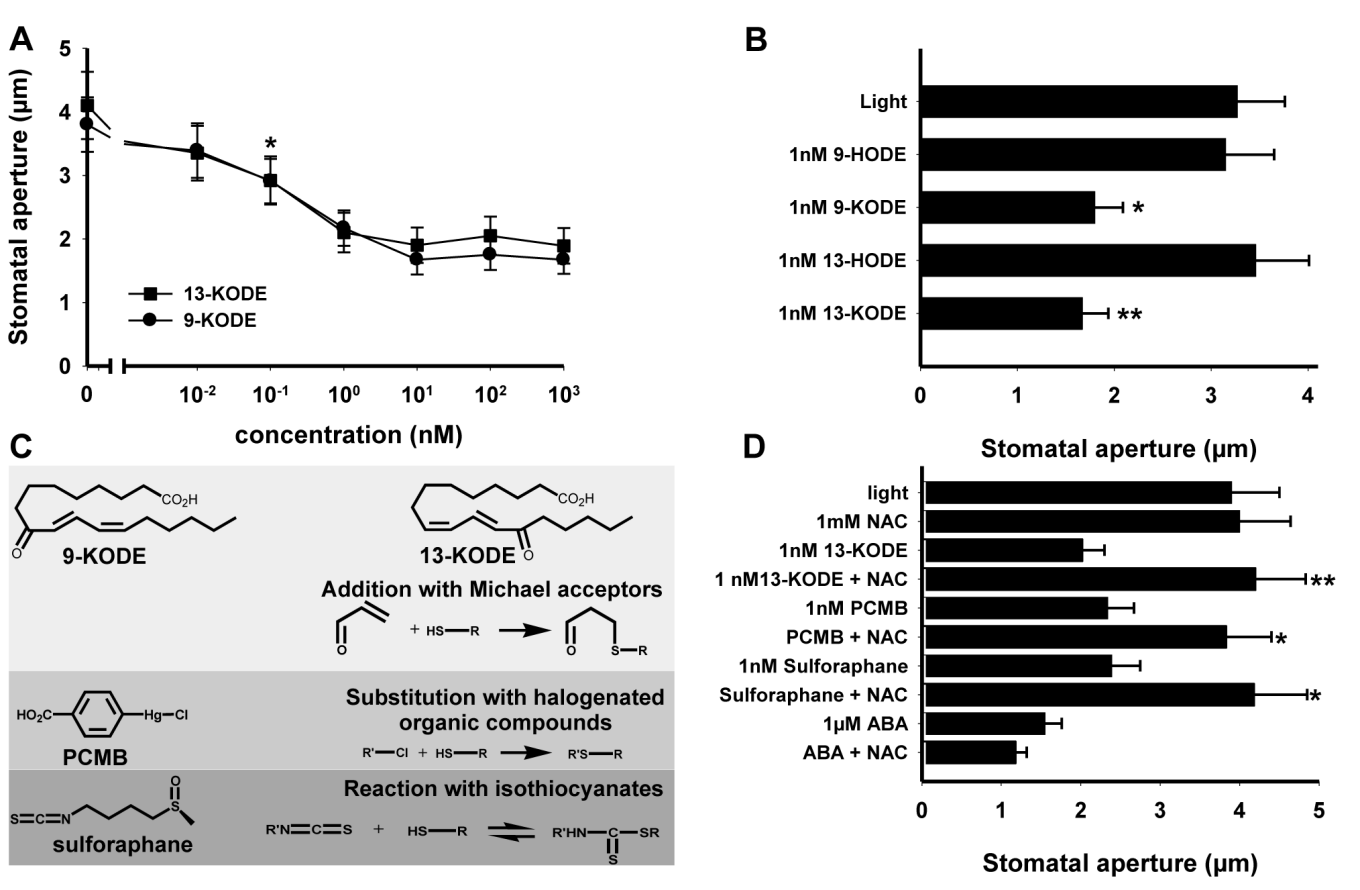
Stomatal closure responses to thiol-reagents. Measurements were performed on Col-0 plants according to the experimental design described in Figure 2, and data represent means ± SD of three independent experiments. (A) Dose response effects of RES oxylipins (9- and 13-KODE). Student's *t* test analysis of means indicates that the effects of 9- and 13-KODE become significant at 0.1 nM. (B) Comparison of the effects of 9- and 13-KODE to their corresponding alcohols 9- and 13-HODE. Values marked with asterisks were significantly different from those of the corresponding alcohols. (C) Structures and reactivity of compounds with thiols. (D) NAC (1 mM) was added to epidermal peels 30 min prior to addition of thiol-reagents or ABA. Marked values were significantly different from those of samples without NAC pre-incubation.

RES oxylipins containing α,β-unsaturated carbonyl groups can result from the metabolization of FAHs by LOX activities (Figure S3) [40],[41]. RES oxylipins are able to add to thiol-containing compounds such as cysteines of proteins or glutathione according to the Michael addition (for review, see [27]) and are produced during plant microbe interactions [22],[26],[42]. We investigated whether the two RES oxylipins, 9-KODE and 13-KODE, show a dose-dependent effect on stomata, finding that both compounds induce stomatal closure already at nanomolar concentrations (Figure 3A).

To test whether the presence of thiol reactive structures was required for the biological activity of 9- and 13-KODE, the ketone group was reduced into nonreactive alcohol. The alcohols, 9- and 13-HODE, were both unable to promote stomatal closure (Figure 3B). Moreover, the effect of different Michael acceptors along with halogenated organic compounds and isothiocyanate was tested. All these molecules are known as thiol reagents and were potent inducers of stomatal closure (Figures 3C, D and S4). Additionally, the thiol-containing compound NAC that inhibited the action of FAHs also blocked the activity of 13-KODE and the other thiol reagents PCMB and sulforaphane, suggesting that reactivity toward thiols was critical for biological activity of these compounds. Conversely, NAC did not prevent ABA-induced stomatal closure (Figure 3D), suggesting that RES oxylipins and ABA do not share a common signaling pathway.

### NAC and 13-HODE Inhibit Bacterial- or flg22- but Not ABA-Induced Stomatal Closure

**Figure 4:**
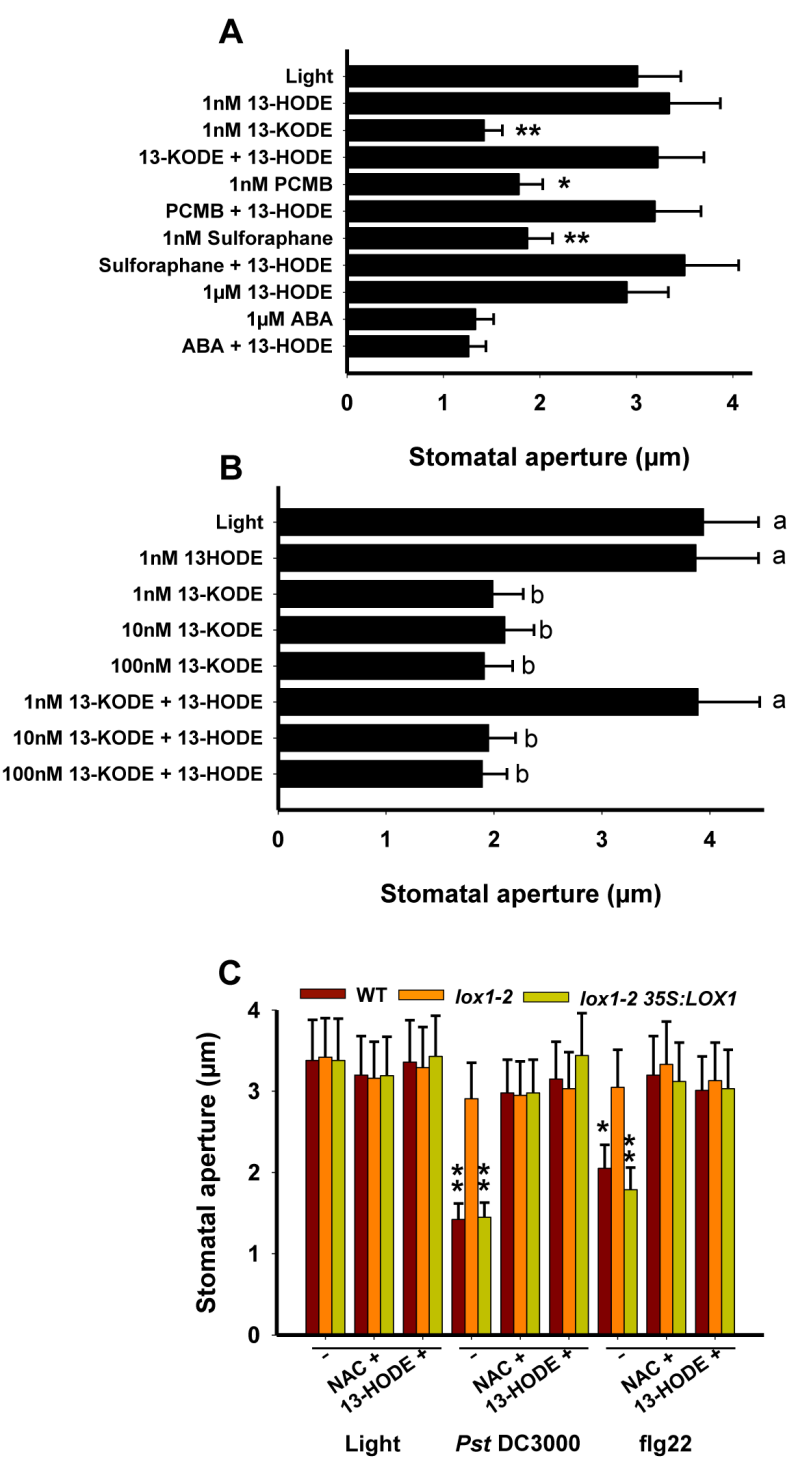
Effects of NAC and 13-HODE on different stimuli inducing stomatal closure. Stomatal aperture measurements were determined on leaf peels from 4–5-wk-old Arabidopsis plants on 2 h light-preincubated epidermal peels after 2.5 h incubation with the following inducers, (A and B) 13-KODE, PCMB, sulforaphane, ABA, or (C) flg22 (5 µM), or after 1 h incubation with the bacterial suspension of *Pst* DC3000 (10^8^ cfu/mL). Pre-treatments with NAC (1 mM) or with 13-HODE (1 nM or 1 µM for ABA treatments) were performed 30 min prior to addition of stomatal closure inducers. Data represent means ± SD of three independent experiments. Asterisks represent significant differences from controls (in (A) samples pre-treated with 13-HODE, in (C) light). In (B) letters represent values not statistically different (a: *F* = 0.0127, *df* = 8, *p* = 0.987; b: *F* = 0.310, *df* = 14, *p* = 0.865).

The biologically inactive alcohols, 9- and 13-HODE, displaying chemical structures close to the corresponding ketones prompted us to verify whether these alcohols could counteract the biological activity of 9- and13-KODE and different thiol reagents. As shown in Figure 4A, pre-incubation with 1 nM 13-HODE abolished the activity of the three thiol reagents (13-KODE, PCMB, and sulforaphane). Similar results were obtained with 9-HODE (unpublished data). However, the inhibitory effect of 13-HODE was overcome by using higher concentrations (10 or 100 nM) of 13-KODE (Figure 4B). On the other hand, stoichiometric concentrations of 13-HODE did not alter the ABA-induced stomatal closure (Figure 4A). Finally, the effects of NAC and 13-HODE were investigated on epidermal peels either inoculated with *Pst* DC3000 or treated with flg22 (Figure 4C). In plants containing a functional *LOX1*, the two compounds abolished stomatal closure induced by both *Pst* and flg22. Together, these data further support the existence of a biotic stress-mediated stomatal response independent of ABA.

### The Bacterial Phytotoxin Coronatine (COR) and Methyl Jasmonate (MJ) Inhibit RES Oxylipin-Induced Stomatal Closure Through Different Mechanisms

**Figure 5:**
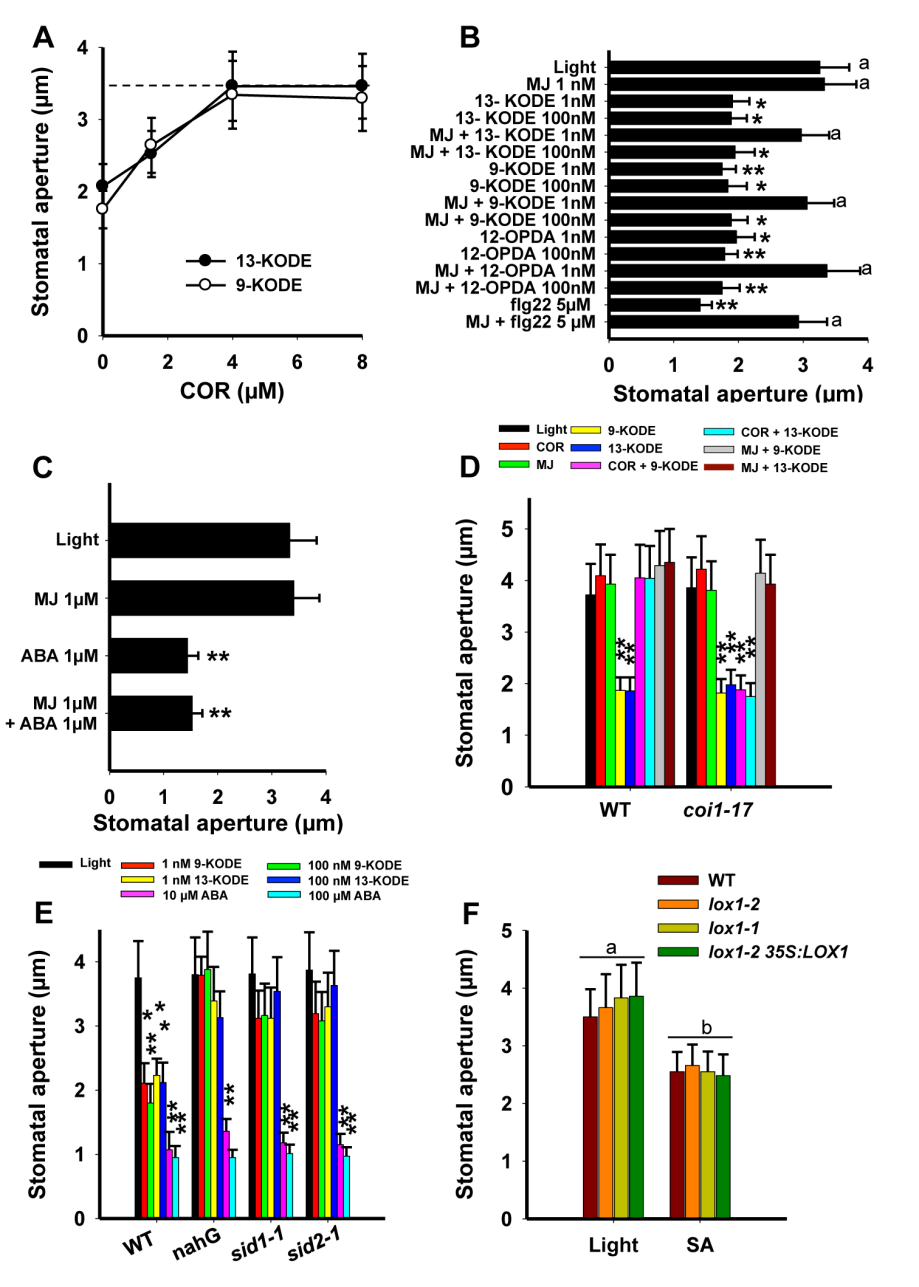
Effects of COR, MJ, and SA on RES oxylipin-, flg22-, and ABA-mediated stomatal closure. (A) Dose response effect of COR pre-treatments on 9/13-KODE (1 nM). COR was added 30 min prior to RES oxylipins according to the experimental design described in Figure 2. The dotted line represents the value of stomatal aperture in samples submitted to light only. (B) MJ was pre-incubated with epidermal peels 30 min prior to addition of RES oxylipins or (C) ABA. Asterisks represent significant differences from controls, and letters represent values not statistically different (a: *F* = 0.520, *df* = 17, *p* = 0.757). (D) Effect of COR (4 µM) or MJ (1 nM) on epidermal peels of the *coi1-17* mutant treated by 9/13-KODE (1 nM). (E) Stomatal aperture measurements were performed on WT (Col-0) nahG transgenic plants, *sid1-1* and *sid2-1* mutant lines after incubations with RES oxylipins or ABA. (F) Effect of SA (100 µM). Letters represent values not statistically different (a: *F* = 0.272, *df* = 11, *p* = 0.844; b: *F* = 0.132, *df* = 11, *p* = 0.939). Data represent means ± SD of three independent experiments.

COR secreted by virulent strains of *Pseudomonas* disables stomatal defense in a COI1-dependent manner and notably inhibits PAMP- and ABA-mediated stomatal closure, subsequently triggering stomatal reopening required for pathogen entry and host colonization [32]. To assess whether COR affects the activity of RES-oxylipin, we pre-treated epidermal peels with the toxin prior to treatment with RES oxylipins. As shown in Figure 5A, COR at 4 µM blocked the effects of both 1 nM 13- and 9-KODE. The COR chemical structure resembles that of JA-Ile, the biologically active form of JA, and enables this phytotoxin to interact with the JA-Ile receptor COI1 to hijack the JA-Ile hormone functions. In an attempt to better understand the biological activity of these chemically related compounds, we examined the action of methyl jasmonate (MJ) on guard cells. In our hands, MJ was unable to promote stomatal closure in a range of concentrations between 1 nM and 100 µM (Figure S4). However, competition experiments of MJ with RES oxylipins (9/13-KODE and 12-OPDA) indicated that pretreatment of epidermal peels with 1 nM MJ reversed the activity of RES oxylipins applied at the same concentration (Figure 5B). At a 100-fold higher concentration of RES oxylipins, this inhibition was no longer observed, suggesting that MJ mediates the inhibition of RES oxylipins activity through a similar mechanism as the alcohols 9/13-HODE (Figure 4). However, in contrast to COR, MJ did not counteract the action of ABA (Figure 5C). In addition, our data establish that although COR and MJ are both able to inhibit the RES oxylipin-mediated stomatal closure, in contrast to COR, the MJ-mediated activity is COI1-independent (Figure 5D).

### Salicylic Acid (SA) Is Required for the RES Oxylipin- but Not For ABA-Induced Stomatal Closure

Bacterial- and PAMP-mediated stomatal closures have been demonstrated to be SA-dependent [32] and SA induces stomatal closure in Arabidopsis [35]. In an attempt to clarify the role of SA in the RES oxylipin- and ABA-signaling cascades, we conducted experiments with the transgenic SA-deficient NahG line, along with the two SA biosynthesis mutant lines, *sid1-1* and *sid2-1* [43],[44]. Results shown in Figure 5E demonstrate that these three lines normally responded to ABA, whereas they were all impaired in their ability to respond to the RES oxylipins 9/13-KODE. In addition, our data also indicate that *lox1* mutant lines were as sensitive to SA (100 µM) as WT and *lox1-2 35S:LOX1* (Figure 5F), demonstrating that exogenously applied SA can complement for *LOX1* deficiency. Together these results support that SA is required to convey the RES oxylipin- but not the ABA-mediated signal leading to stomatal closure.

### The Protein Kinase OST1 Does Not Participate in the Flagellin-Mediated Signaling Cascade

**Figure 6:**
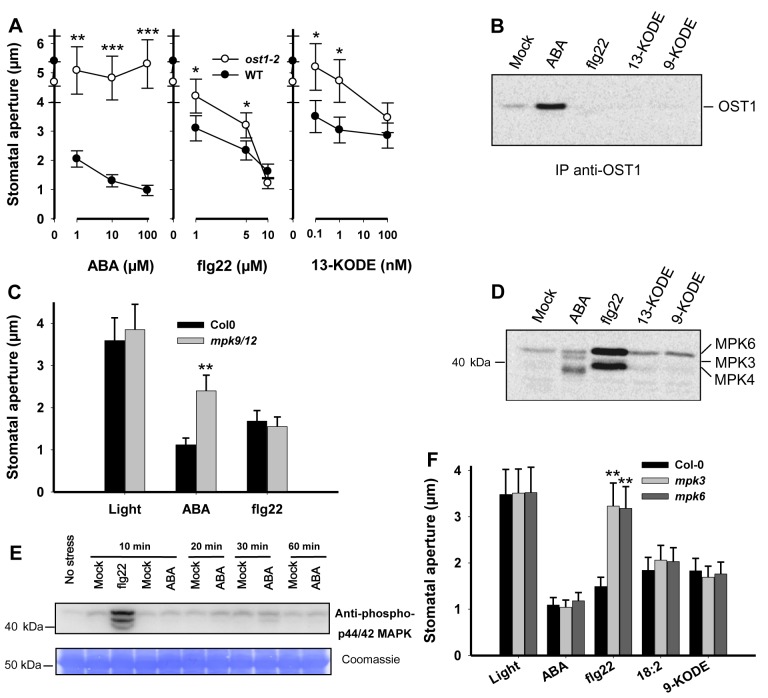
Involvement of OST1 and MAPKs in biotic- and ABA-mediated stomatal closure. (A) Dose response effects of ABA, flg22, and 13-KODE were performed on WT (Ler) and *ost1-2* mutant plants according to the experimental design described in Figure 2, and data represent means ± SD of three independent experiments. (B) OST1 activity in Col-0 suspension cells was analyzed at 10 min by in-gel kinase assay after the treatments: ABA (30 µM), flg22 (100 nM), and 9- and 13-KODE (100 nM). 0.1% ethanol in water (mock). Kinase activity was estimated on proteins immunoprecipitated with anti-SnRK2-6 (OST1) antibody. The gel is typical of three independent experiments. (C) Stomatal aperture measurements were performed on WT (Col-0) and *mpk9-1/12-1* mutant plants after incubation with ABA (10 µM) and flg22 (5 µM). (D) Protein kinase activities of Col-0 suspension cells were analyzed at 10 min by in-gel kinase assay after the treatments: ABA (30 µM), flg22 (100 nM), and 9- and 13-KODE (100 nM). Kinase activity was estimated on 20 µg of crude protein extracts, and the gel is typical of three independent experiments. (E) Typical immunoblot of three independent experiments performed with total proteins extracted from 2-wk-old plants (Col-0) either treated with ABA (100 µM) or flg22 (1 µM). Mock treatments were 0.1% ethanol in water for ABA and water for flg22. MAPKs were detected (upper panel) with the rabbit anti-phospho-p44/42 MAPK (Erk1/2) (Thr202/Tyr204) monoclonal antibody. Coomassie staining of proteins is shown for loading control (lower panel). (F) Stomatal aperture measurements were performed on WT (Col-0), *mpk3*, and *mpk6* mutant plants after incubation with ABA (10 µM), flg22 (5 µM), linoleic acid (18∶2, 100 nM), and 9-KODE (1 nM).

Many steps involved in the ABA-dependent signaling pathway are now well documented [45],[46]. More recently, ABA has also been described as a key player in pathogen- and PAMP-induced stomatal responses [33]. It has been demonstrated that the Arabidopsis mutants *aba3-1* and *ost1-2*, which are defective in the hormone biosynthesis and signaling, respectively, were both no longer able to close stomata in response to either *Pst* or flg22. In order to clarify the function of ABA signaling in pathogen-induced stomatal closure, we first compared the effects of ABA, flg22, and 13-KODE on stomata of the mutant *ost1-2*. As previously reported, the *ost1-2* mutant does not close stomata in response to ABA even at very high concentrations (Figure 6A). However, *ost1-2* plants remained responsive to both flg22 and 13-KODE, albeit at higher doses than WT plants. The stomatal behavior of the *aba2-1* mutant, which is defective in ABA production, was also assessed and showed a similar response profile as the *ost1-2* mutant (Figure S5).

In order to get further insights into the signaling steps triggered by the three compounds, we have assessed the activation of OST1 on Arabidopsis cell suspensions. In-gel kinase assays (Figure 6B) of immunoprecipitated OST1 showed that in accordance with previous work [47]–[49], ABA clearly activated OST1 kinase. In contrast, flg22 and both RES oxylipins 9- and 13-KODE were unable to activate OST1. This is consistent with the hypothesis that different signaling pathways can lead to stomatal closure, but that the optimization of the guard cell response to biotic stress requires ABA-dependent mechanisms.

### Distinct MAP Kinase Pathways Mediate ABA and Biotic Stress Signals

Apart from OST1, a number of other protein kinases have been identified to function in ABA signal transduction, including mitogen-activated protein kinases (MAPKs), which play a prominent role in both biotic and abiotic stress signaling [50],[51]. MPK9 and MPK12 were shown to play an important role in ABA-induced closure of stomata [29]. To clarify the role of these MAPKs in ABA and flg22 signaling, we tested *mpk9-1/12-1* double mutant plants, which were reported to be impaired in ROS-mediated ABA signaling [29]. Double mutant plants displayed a clear defect in ABA-induced stomatal closure, but were as responsive to flg22 as wild-type plants (Figure 6C).

To further investigate the role of protein kinases in response to ABA and biotic stress, we carried out kinase assays on cells treated with ABA, flg22, and 9- or 13-KODE. In-gel kinase assays showed flg22-mediated activation of three protein kinases (Figure 6D) that were previously identified as MPK3, MPK4, and MPK6 [52]. Immunoblotting using the anti-phospho-p44/42 MAPK antibody confirmed the presence of these activated MAPKs in seedlings exposed to flg22 (Figure 6E). However, none of these MAPKs were activated in response to ABA (Figures 6E), the kinase activities stimulated in ABA-treated cells corresponding to OST1 and other SnRK2s [48]. In order to verify the role of these kinases in the ABA- and PAMP-mediated signaling cascades, the behavior of *mpk3* and *mpk6* knockout mutants was investigated. As shown in Figure 6F, stomata of *mpk3* and *mpk6* responded normally to ABA, 18∶2 and 9-KODE, whereas their response to flg22 was strongly impaired demonstrating that different sets of MAPKs are required to convey signals generated by the action of ABA and biotic stress and suggesting that RES oxylipin production and action occur downstream of MPK3/6.

### Distinct Pathways Mediate ABA and Bacterial Early Gene Expression

To further assess whether early pathogen-mediated signaling cascade depended on ABA, plantlets were submitted either to ABA or to *Pst* DC3000 or to *Pst* followed by ABA sprays and sampled over a time scale corresponding to the pathogen-induced stomatal closure. As shown in Figure S6, transcript levels of the three ABA-specific genes, *RD29b*, *ABI1*, and *ABI2*, (Methods S1), significantly increased as soon as 30 min after application of the hormone, whereas transcript levels of these genes did not increase in plants treated with *Pst* DC3000. In addition, pretreatment with *Pst* did not modify the response of plants to ABA. Together, these results suggest that during the early phase of the plant–pathogen interaction when stomatal closure takes place, ABA is not produced.

## Discussion

### LOX1 Activity Controls Stomatal Closure During the Early Stages of Plant–*Pst* Interactions

In the present work, we show that a defect in *LOX1* compromises the ability of plants to close stomata in response to both virulent and avirulent strains of *Pst* or the PAMP flg22 (Figure 1). Impairment in ABA responsiveness, which could have explained the *lox1* mutant phenotype, was ruled out since mutant and WT lines were similarly able to close stomata upon ABA treatment (Figure 1). On the other hand, involvement of oxylipins arising from LOX1 activity in controlling stomatal access of bacteria into plants was clearly established (Figure 1). Induction of stomatal closure by LOX substrates (PUFAs) or LOX reaction products (FAHs and KOD(T)E) was demonstrated (Figures 2, 3, and S4). On the other hand, Arabidopsis mutants lacking *LOX1* were no longer able to respond to PUFAs (Figure 2), while they still remained responsive to FAHs (unpublished data) and 9/13-KODE (Figure S7A), indicating that absence of LOX1 activity can be rescued not only by FAHs but also by their metabolites. In line with this result, it is noteworthy that pretreatment of the *lox1-2* mutant line with the 9-specific LOX compound 9-KODE (Methods S1), partially restores resistance of LOX1-deficient lines against *Pst* DC3000 (Figure S7B), confirming that LOX1 controls stomatal defense.

Dose-response curves of linoleic acid and the two FAHs (9- and 13-HPODE) for stomatal closure showed that these compounds close stomata at concentrations several orders of magnitude lower than ABA (Figures 2 and S4). Our data also demonstrate that the efficiency of oxylipins to close stomata is not position-specific, since 9- and 13-HPODE had the same biological activity and that trienic fatty acids were as active as dienic ones (Figure 2). To better understand the mechanisms responsible for the biological activity of FAHs, their efficiency was compared to that of H_2_O_2_. FAHs were remarkably more active than H_2_O_2_ and tBuOOH (Figure 2). To explain this discrepancy, we postulate that oxidative stress potentially generated by the peroxides might target different signaling components. Unlike H_2_O_2_ or tBuOOH, the more hydrophobic FAHs might access and oxidize critical residues in hydrophobic sites of key signaling proteins.

Thiol-containing compounds can protect cells against oxidative injury by scavenging reactive oxygen species (ROS) or by conjugating to reactive electrophile species (RES) often generated from FAHs [27]. NAC, used in the present work for its rather weak antioxidant property [38], did not inhibit the H_2_O_2_- or tBuOOH-induced responses, but it strongly compromised FAH responses (Figure 2). Hence, it is likely that inhibition of FAH activity by NAC was rather due to its nucleophilic properties. Downstream of FAHs, those oxylipins containing α,β-unsaturated carbonyl structures are powerful RES able to add to thiol-containing substances [25]–[27],[39]. Consequently, the activity of FAHs on stomata can rather be explained by their metabolization into RES-oxylipins (Figure S3). In good agreement with this hypothesis, the two RES oxylipins 9- and 13-KODE displayed equivalent and very strong activity on guard cells (Figure 3). Five additional RES oxylipins including trienic fatty acid ketones (9- and 13-KOTE), two cyclopentenone derivatives (12-OPDA and ProstaglandinA2), and the aldehyde 4-HNE that can be generated by decomposition of FAHs all displayed strong biological activity (Figure S4).

Among these oxylipins, the JA precursor 12-OPDA displayed a strong activity on stomata, whereas MJ was inactive in a range of concentrations between 1 nM and 100 µM (Figure S4). The ability of MJ to induce stomatal closure is still controversial ([53]–[55] and this work), but very recent results show that MJ is active only when ABA endogenous levels reach a critical threshold [56]. In the ABA-deficient line *aba2-2* or in plants treated by fluridon, an inhibitor of ABA biosynthesis, MJ was no longer able to promote stomatal closure, indicating that MJ activity on guard cells is intimately dependent on the physiological status of plants. Hence, culture conditions used to produce the biological material can strongly influence this status and modify the ability of plant to respond to MJ.

The α,β-unsaturated carbonyl structure in oxylipins, as soft electrophiles, preferentially add to soft nucleophiles, which are cysteine residues in GSH or proteins [27]. Reducing the RES oxylipins, 9-, and 13-KODE into their corresponding alcohols 9- and 13-HODE, the latter being not electrophilic, has allowed us to assess the involvement of this putative mechanism. Given that both alcohols were inactive (Figure 3), reactivity of 9- and 13-KODE toward thiols could explain their biological activity. This hypothesis was confirmed by the fact that a large set of known thiol-reagents, including different natural and artificial Michael acceptors, several halogenated organic compounds, and one isothiocyanate, all promoted stomatal closure at nanomolar concentrations (Figures 3C, D and S4). Altogether, these results strongly suggest the involvement of a thiol-containing target in the RES oxylipin-mediated cascade.

At stoichiometric concentrations, both the fatty acid alcohols 9/13-HODE and MJ were found to block the biological activity of RES oxylipins but not that of ABA (Figures 4 and 5). On the other hand, at concentrations above 1 µM, the *Pseudomonas* phytotoxin COR disabled both RES oxylipin and ABA activities ([32] and Figure 5). These results suggest that the COR-mediated inhibition of RES oxylipin activity is COI1-dependent, whereas fatty acid alcohols and MJ inhibit this activity by means of another mechanism. This hypothesis is further supported by results shown in Figure 5D, which indicate that, on the mutant *coi1-17*, COR no longer exerts inhibition of the ketone effects, whereas MJ remains active. Neither NAC, 13-HODE, nor MJ inhibited the activity of ABA (Figures 4 and 5), suggesting that RES oxylipins do not share a common signaling mechanism with ABA. Considering that stomatal closure induced by inoculation with *Pst* or treatment with flg22 were inhibited by NAC or 13-HODE (Figure 4), it can be concluded that the early stomatal response to pathogens is not under the control of ABA but depends on LOX1-derived RES oxylipins. According to all these data, it is tempting to speculate that thiol-reactive compounds such as RES-oxylipins generated in response to biotic stress would react on guard-cell-specific target(s) bearing a critical cysteine residue buried in a hydrophobic cavity. This covalent binding (alkylation) would then trigger stomatal closure. Competition with thiol-containing compounds such as NAC or the presence of FAH or RES oxylipin metabolites such as 9/13-HODE and jasmonates that could fit into the target cavity without reacting with the critical cysteine residues would prevent the target alkylation and thereby inhibit the biological process. The equal activity of 9- and 13-oxylipins on stomata suggests that these compounds are perceived by the same guard cell target(s). The 9- and 13-LOXs could both contribute to production of metabolites regulating stomatal movements, however in mutants lacking LOX1 activity, (i) PUFAs were not able to rescue stomatal function and (ii) bacteria were unable to trigger stomatal closure. Hence, LOX1 exhibits a specific function for which no other LOXs can be substitutes. Consistent with this assumption, it is clearly established that mutation in *LOX6*, the 13-LOX encoding gene expressed in guard cells, did not impair stomatal responses to flg22, linoleic acid, or 9-KODE (Figure S2).

Our examination of the SA function in the stomatal response indicated that the synthesis of SA is required to convey the RES oxylipin (9/13-KODE) signal but not that of ABA and SA acts downstream of these RES oxylipins (Figure 5). Hence it can be concluded that SA participates in the RES oxylipin-mediated signaling cascade in accordance with previous results indicating that PAMP- or *Pst*-regulated stomatal closure was SA-dependent [32] but SA is not an intermediate of the ABA-dependent pathway (Figure 5). Again, these results further support the conclusion that ABA does not act as a regulator of the biotic stress-dependent signal in guard cells.

### Bacterial-Induced Stomatal Closure Is ABA-Independent

A major breakthrough in the understanding of the plant innate immune response against bacterial pathogens was the finding that stomatal closure was a key part of this process preventing bacterial entry in leaves [33]. This work also suggested that PAMP-induced stomatal closure required ABA biosynthesis and components of the ABA signal transduction pathway such as the protein kinase OST1 and nitric oxide biosynthesis.

Our data argue in favor of the existence of an ABA-independent guard cell signaling cascade controlling stomatal closure during the early steps of plant-microbe interaction. First, the ability of *lox1* mutant lines to respond to ABA strongly suggested that LOX1 did not act downstream of the hormone (Figure 1). Second, in contrast to ABA, neither flg22 nor the RES oxylipins 9- and 13-KODE could activate OST1 kinase in Arabidopsis cells (Figure 6), suggesting that different early signaling pathways are employed for ABA and flg22 or RES oxylipins. Consistent with these results, the mutant *ost1-2*, which is insensitive to ABA, still responded to flg22 or 13-KODE (Figure 6), albeit responses were less intense than in WT. Similarly, the stomatal responses of the *aba2-1* mutant, which is impaired in ABA synthesis, showed a marked decrease in sensitivity not only to ABA but also to flg22 and 13-KODE (Figure S5). Overall, our results strongly suggest that although ABA is not the major regulator of bacterial and PAMP-induced stomatal closure, it contributes to modulate stomatal responses to biotic stress. In a recent genetic screen, Zeng et al. [35] identified Arabidopsis mutants named scord (susceptible to coronatine-deficient *Pst* DC3000) that were compromised in their ability to close stomata in response to bacteria. Interestingly, a majority of these mutants showed normal closure in response to ABA, confirming our results that ABA does not directly control bacterial-induced stomatal closure. Stomatal closure requires the presence in guard cell membranes of effector proteins that control osmotic pressure. Their transcription and/or posttranslational regulation are known to mainly depend on ABA in an OST1-mediated pathway [57]. To explain that mutants affected in ABA synthesis or regulation remained responsive to biotic stress, it must be assumed that still unidentified components also regulate this ultimate step. However, in these mutants, the stomatal closure machinery is likely less efficient, explaining the lower sensitivity of such mutants to biotic stress. Interestingly, the activation of one of these effectors, the slow-type anion channel SLAC1 [58], is required in both ABA- and biotic stress-mediated responses (Figure S9), suggesting that these pathways converge at the level of the ion channels controlling the osmotic pressure of the guard cell necessary to regulate stomatal aperture.

Numerous MAPK cascades have been reported to convey diverse environmental signals [50],[51]. Both MAP kinases MPK9 and MPK12 were identified as critical steps of the guard cell response to ABA, and the *mpk9-1/12-1* double mutant is compromised in ABA-induced stomatal closure [29]. However, this mutant responded normally to flg22 (Figure 6), further supporting that distinct ABA- and flg22-mediated pathways exist in guard cells. The two well-characterized MAPKs, MPK3 and MPK6, that are key response elements in both abiotic and biotic stress signaling have also been implicated in ABA signal transduction. *MPK3* antisense plants were shown to be partially impaired in ABA-promoted inhibition of stomatal opening, while they responded normally to the hormone in stomatal closure [59]. However, these plants were compromised in their ability to close stomata in response to *Xanthomonas campestris* inoculation [60]. MPK6 was also reported to mediate ABA- and sugar-regulated seed germination [61] but also responses to other stresses such as pathogen infection, cold and salt stress, and wounding [62]–[65]. Our work demonstrated that flg22 but not ABA activated MPK3, MPK4, and MPK6 early after treatment (Figure 6). A role of MPK3 and MPK6 in flg22-induced stomatal closure was subsequently confirmed by testing *mpk3* and *mpk6* knockout lines. The fact that both mutants were compromised in the flg22-mediated stomatal closure (Figure 6) suggests that the two MAP kinases do not play a redundant role in this process and agrees with recent findings that these two kinases both play an important but distinct role in plant immunity and signaling [66],[67]. Overall, our results demonstrate that MPK3 and MPK6 are involved in the flagellin, but not in the ABA guard cell signaling cascade. The fact that linoleic acid (18∶2) and 9-KODE were as active on WT as on *mpk3* or *mpk6* mutants suggests that LOX1-mediated synthesis of oxylipins is downstream of MPK3/6, although it cannot be ruled out that these two steps are independent. The involvement of these MAPK activities as early specific steps of the flg22-dependent signaling cascade provides another argument supporting the existence of an ABA-independent mechanism leading to stomatal closure.

Finally, gene expression studies of plantlets that were treated with ABA or *Pst* DC3000 showed that *RD29b*, *ABI1*, and *ABI2* transcript levels rapidly increased only in response to ABA but not after *Pst* inoculation (Figure S6). Additionally, given that plants first treated with *Pst* and then sprayed with ABA displayed identical responses as those observed in plants treated by ABA only, it can be concluded that ABA is not produced during the very early stage of the interaction with bacteria.

**Figure 7:**
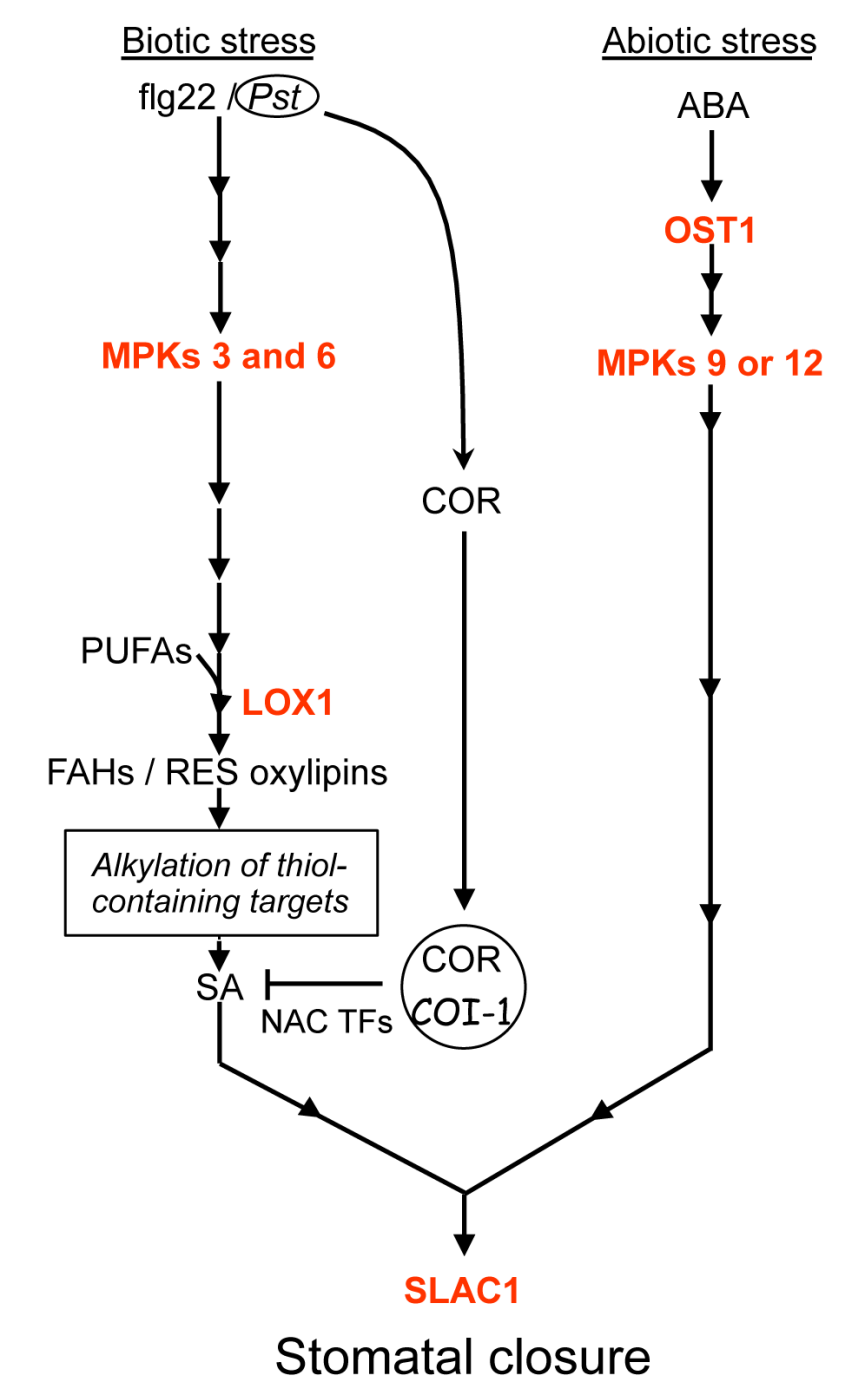
Working model showing signaling components involved in bacterium- or flg22- and ABA-mediated pathways that lead to stomatal closure in the Arabidopsis guard cell. In red are the steps whose activity has been demonstrated in this work by genetic approaches. Downstream of biotic stimuli (*Pst* or flg22), both MPK3 and MPK6 are required to activate production of LOX1-dependent oxylipins such as FAHs (9-HPOD(T)E) and/or reactive ketones (9-KOD(T)E). Our pharmacological data strongly suggest that the latter compounds modulate the activity of putative thiol-containing targets responsible for biotic signal transduction. Downstream of this step, SA is required to convey the biotic stress signal. In a COI1-dependent manner, COR inhibits the accumulation of SA through the activity of three NAC transcription factors and orchestrates the stomatal reopening [74]. On the other hand, the ABA-dependent pathway that is triggered upon abiotic stress requires the protein kinases OST1 and MPK9 or MPK12. As one of the final effectors required for the regulation of osmotic pressure in guard cells, activation of the slow-type anion channel SLAC1 is necessary for stomatal closure in response to both biotic stress and ABA. Not depicted in this model are the data provided by this work and supporting the involvement of ABA in the guard cell potentiation required for optimizing responses to biotic stress.

Overall, our data suggest that early signals produced upon challenging of plants with ABA and pathogens are different. As depicted in the proposed model (Figure 7), whereas the response to ABA is transduced principally by OST1 and MPK9/12, microbial signals are conveyed by MPK3/6, LOX1-induced RES oxylipin derivatives, and SA. Our work also provides evidence that ABA is essential to potentiate guard cells to enable them to properly respond to biotic stimuli, and further studies are necessary to characterize these steps and their role in the stomatal responses to other signals. Finally, the present results shed new light on the function of oxylipins in the plant immune response.

## Materials and Methods

### Statistics

All experiments were performed in triplicates, and statistical differences of means were analyzed with the Student's *t* test with the following symbols for the classes of probability: * *p*<0.05, ** *p*<0.01, and *** *p*<0.001. To compare more than two means, the ANOVA test was performed and letters have been used to mark statistically identical groups of means. The number of degrees of freedom (*df*s), the value of the *F* statistic and the *p* value are given in the legend of figures for each group of means.

### Plant Materials

The following *Arabidopsis thaliana* transgenic and mutant lines used in this study were derived from ecotype Columbia (Col-0), *lox1-2 35S:LOX1* (see below), *lox1-1*, *lox1-2* [7], *lox6-1* (see below), *aba2-1*, *mpk3*, *mpk6* [67], *mpk9-1/12-1* [29], nahG [68], *slac1-3* [58], *sid1-1* [44], *sid2-1* [43], and *coi1-17* [69], whereas the *ost1-2* line derived from *Landsberg erecta* (Ler). Specific growth conditions are specified below for each type of experiment. *coi1-17* mutant plants were selected from an heterozygote population using root sensitivity to 10 µM MJ according to [69].

### Transgene Construction and Complementation of the *lox1-2* Mutant Line

The *Escherichia coli* strain TOP10 (Invitrogen) was used for cloning and propagation of the different vectors. *Agrobacterium tumefaciens* C58 strain transformation was performed by electroporation and was used in all plant transformation experiments. The full-length cDNA clone of *LOX1* gene (accession number BT010358) provided by the Nottingham Arabidopsis Stock Center (http://arabidopsis.info) was used for this study. PCR amplification was performed with the cDNA containing vector as template and the primer couple: forward, 5′-GGGGACAAGTTTGTACAAAAAGCAGGCTTCGAAAACCTGTATTTTCAGGGAATGTTCGGAGAACTTAGGGATCTG-3′ and reverse, 5′-GGGGACCACTTTGTACAAGAAAGCTGGGTTTCAGATAGAGACGCTATTTGGAAT-3′. The amplicon was subcloned into the GATEWAY donor vector pDONRZeo (Invitrogen) using the BP clonase II (Invitrogen). The full-length cDNA was subcloned in the destination vector pMDC32 kindly provided by Mark Curtis (Institute of Plant Biology and Zürich-Basel Plant Science Centre, University of Zürich, Switzerland) using the LR clonase (Invitrogen). The homozygous T-DNA line *lox1-2* was transformed with empty vector *pMDC32* or *pMDC32:LOX1* using flower dipping method. T1 plants were screened on germination medium supplemented with Hygromycine (30 mg/L). The T-DNA presence was monitored by PCR analysis using the primer couple: forward, 5′-GCGCGATTGCTGATCCCC-3′ and reverse, 5′- GCCCTCGGACGAGTGCTG-3′. The expression of *LOX1* in the transgenic lines was measured by semi-quantitative RT-PCR using the primer couples: (*LOX1*) forward, 5′-AGACTATCCTTACGCAGTGGA-3′ and reverse 5′-TGCCGGTGACTCCGCCTTC-3′, and (*EF1*) forward, 5′-TACCTCCCAGGCTGATTGTG-3′ and reverse, 5′-TCTGACCAGGGTGGTTCATG-3′. Seven *lox1-2:pMDC32:LOX1* independent lines were retrieved after the screening, T2. Progeny analysis was performed and lines with a single T-DNA locus were selected for further experiments.

### Chemicals and Synthesis of 9- and 13-HODE

Oxylipins and other chemicals used in this study are given in Table S2. Flg22 was kindly provided by Dr. Laurent Noël (Laboratoire des Interactions Plantes-Microorganismes CNRS/INRA Toulouse, France). The alcohols, 9-HODE, (10*E*,12*Z*)-9-hydroxy-10,12-octadecadienoic acid and 13-HODE, (9*Z*,11*E*)-13-hydroxy-9,11-octadecadienoic acid were produced by sodium borohydride reduction of the corresponding ketones according to the following procedure: 10 µL of 10 mM 9- or 13-KODE ethanolic solution was added to 20 µL of 5% (w/v) NaBH_4_ in 0.2 N NaOH and incubated 5 min at room temperature. After addition of 100 µL of 0.5 M potassium acetate pH 4.0, products were extracted 3 times with 200 µL of the solvent mixture hexane/diethyl ether, 70/30 (v/v). The organic phase was then evaporated to dryness under a nitrogen stream, and the residue was dissolved in absolute ethanol. Purity of compounds was controlled by HPLC chromatography according to Montillet et al. [70]. The chromatographic profiles were recorded at 234 and 280 nm for alcohol and ketone detections, respectively. None of the alcohol preparations were contaminated with a detectable amount of ketone.

### *Pseudomonas syringae* Growth Assay

Plants were grown on soil in a controlled environment chamber under an 8 h light regime (150–200 µE·m^−2^·s^−1^) at 22°C and 65% relative humidity. *Pst* DC3000 was grown for 24 h at 28°C on NYGA solid medium supplemented with 50 µg/mL Rifampicin. For *in planta* bacterial growth assays, 4-wk-old plants were spray-inoculated with bacterial suspensions at 5·10^7^ cfu/mL in 10 mM MgCl_2_ with 0.03% (v/v) Silwet L-77 (Lehle Seeds). *In planta* bacterial titers were determined at day 3 by shaking leaf discs in 10 mM MgCl_2_ with 0.01% (v/v) Silwet L-77 at 28°C for 1 h as previously described [71]. At least five plants per genotype were used for each sampling.

### Measurements of Stomatal Aperture

After 3 d of stratification of seeds at 4°C, wild-type, mutant, and transgenic lines of *A. thaliana* were grown on soil in a plant growth chamber with an 8 h light period (250 µE·m^−2^·s^−1^) at 23°C and a 16 h dark period at 19°C, and relative humidity of 75%. The abaxial side of leaves of 4- to 5-wk-old plants was stuck on cover slips and peeled. Samples were then placed in Petri dishes containing 10 mM MES/Tris pH 6.0, 30 mM KCl, and 1 mM CaCl_2_ (working buffer). After 30 min in darkness, epidermal peels (except dark controls) were transferred for 2 h under light (300 µE·m^−2^·s^−1^) at 22°C in order to assure that most stomata were open before treatments. For promotion of closure assays, stock solutions of compounds (in water or ethanol as indicated in the figures) were directly diluted in the working buffer in contact with epidermal peels. Stock solutions prepared in ethanol were diluted so that the final solvent concentration did not exceed 0.1% (v/v). *Pst* DC3000 and *Pst* DC3000 *AvrRpm1* were grown for 18 h in a rotating incubator at 30°C and 200 rpm in liquid LB medium supplemented with 50 µg/mL of Rifampicin and 50 µg/mL of Rifampicin plus 10 µg/mL of tetracycline, respectively. For treatments, bacterial suspensions were centrifuged and resuspended in the working buffer at a final concentration of 10^8^ cfu/mL. After treatments, samples were further incubated under light for 2.5 h (or 1 h for treatments with bacteria) before stomatal aperture measurements according to Merlot et al. [72]. To avoid bias results, blind experiments were performed so that experimenters in charge of stomatal measurements were not informed of the evaluated genotypes or treatments. Values reported are the means of at least three independent experiments, for which 60 aperture widths were measured each time. Error bars represent standard deviations of the means.

### Immunoblotting

*Arabidopsis thaliana* Col-0 seedlings were grown in liquid MS medium (SIGMA) for 14 d in a growth chamber (24°C, 16 h photoperiod). Seedlings were treated with water (mock) or 1 µM flg22 for 10 min or with 0.1% ethanol (mock) or 100 µM ABA for 10, 20, 30, or 60 min. For protein extraction, ground samples were resuspended in SDS-PAGE loading buffer, boiled, and centrifuged at 8,000 *g* for 2 min. Supernatants, containing total protein extracts, were separated on 10% SDS-PAGE gels and transferred to PVDF membranes. After blocking with 5% BSA in TBST, membranes were probed with 1/5,000 anti-phospho-p44/42 MAPK (Erk1/2) (Thr202/Tyr204) rabbit monoclonal antibody (Cell Signaling) followed with 1/10,000 horseradish peroxidase (HRP)-conjugated anti-rabbit IgG antibody (SIGMA). HRP activity was detected using the ECL Plus reagent kit (GE Healthcare) and the GeneGnome imaging system (Syngene). Coomassie blue staining of blots was carried out for protein visualization.

### In-Gel Kinase Assay

*Arabidopsis thaliana* cell suspension (T87 line, Columbia ecotype) was cultured as previously described [73]. Cells were treated with 30 µM ABA, 100 nM flg22, 100 nM 13-KODE or 9-KODE, or 0.1% ethanol (mock) for 10 min. Protein extraction, immunoprecipitation with anti-SnRK2.6 (OST1) specific antibody [45] followed by in-gel kinase assay were performed as previously described [42].

### Accession Numbers

The accession numbers for the genes discussed in this article are as follows: *LOX1* (At1g55020), *LOX2* (At3g45140), *LOX3* (At1g17420), *LOX4* (At1g72520), *LOX5* (At3g22400), *LOX6* (At1g67560), *OST1* (At4g33950), *MPK3* (At3g45640), *MPK6* (At2g43790), *MPK4* (At4g01370), (At3g18040), *MPK9* (At3g18040), *MPK12* (At2g46070), *ABA2* (At1g52340), *ABI1* (At4g26080), *ABI2* (At5g57050), *RD29b* (At5g52300), *SLAC1* (At1g12480), *COI1* (At2g39940), *SID1* (At1g74710), and *SID2* (At4g39030).

## Supporting Information

Figure S1 *LOX* gene expression analyses in leaf cells of Arabidopsis. (A) Data were extracted from microarray expression analyses published by Leonhardt et al. [30]. Expression levels of each gene in guard cells (GC) and mesophyll cells (MC) were normalized to 5S rRNA subunit. (B) RT-PCR of *LOX* expressed in leaves with RNA extracted from highly purified guard cell (GC) and mesophyll cell (MC) protoplasts. The *Actin2* gene was used as a control.
(TIF)

Figure S2 Stomatal closure responses of the *lox6-1* mutant line to flg22, fatty acids, and 9-KODE. (A) Scheme of *LOX6* genomic locus with exons represented as black boxes. The position of the T-DNA insertion is indicated. (B) RT-PCR of RNA isolated from leaves. Gene At5g21430 encoding the subunit U of NADH dehydrogenase-like complex (NDH-U) was used to normalize transcript levels in each sample. Gene-specific primer sets used for the evaluation of RNA are shown in Table S1. (C) Stomatal aperture measurements were performed on 2 h light-preincubated epidermal peels of 4–5-wk-old WT (Col-0) and *lox6-1* mutant plants after 2.5 h incubation with flg22 (5 µM) linoleic acid (18∶2, 100 nM) and 9-KODE (1 nM).
(TIF)

Figure S3 Simplified oxylipin pathway. Only oxylipins are described whose activity has been assessed on stomata of Arabidopsis. Fatty acid hydroperoxides (FAHs: 9/13-HPOD(T)E) and ketones (9/13-KOD(T)E) are synthesized in Arabidopsis by 9-specific (LOX1 and LOX5) and 13-specific LOXs (LOX2, LOX3, LOX4, and LOX6), whereas the alcohols (9/13-HOD(T)E) result from reduction of FAHs by reductases. Metabolization of 13-HPOTE by allene oxyde synthase (AOS) and allene oxyde cyclase (AOC) leads to the formation of the cyclopentenone (12-OPDA) and jasmonates (JAs). RES oxylipins are marked with an asterisk. Full names of these metabolites are given in Table S2.
(TIF)

Figure S4 Stomatal closure responses to thiol-reagents and MJ. (A) Structures of natural and synthetic compounds inducing stomatal closure when incubated for 2.5 h on epidermal peels from 4–5-wk-old Col-0 plants. (B) Raw data showing the effect of compounds tested at a concentration of 1 nM (gray bars). For comparison, MJ has been tested at 100 µM (dark gray bar). Data represent means ± SD of three independent experiments, and values marked with identical letters were not statistically different.
(TIF)

Figure S5 Comparative effects of ABA, flg22, and 13-KODE on stomata of the Arabidopsis *aba2-1* mutant line defective in ABA synthesis. Dose response effects of ABA, flg22, and 13-KODE were performed on 2 h light-preincubated epidermal peels of 4–5-wk-old mutant plants, *abi1-1* and their corresponding WT ecotype Col-0, after 2.5 h incubation with compounds. Data represent means ± SD of three independent experiments.
(TIF)

Figure S6 Early gene expression in response to ABA and *Pst* DC3000. Three-week-old plants Col-0 were sprayed with ABA (100 µM), *Pst* DC3000 suspensions (10^9^ cfu/mL), *Pst* DC3000 followed by ABA 30 min later or Mock (0.1%, v/v ethanol in water). After treatments, plants were sampled at 0.5 and 1 h for cDNA synthesis and quantitative qRT-PCR. The transcript levels of ABA-responsive genes, *RD29b* (At5g52300), *ABI1* (At4g26080), and *ABI2* (At5g57050), were normalized using *EF1* (At5g60390), and data represent means ± SD of three independent experiments. Values marked with identical letters were not statistically different.
(TIF)

Figure S7 Effects of RES oxylipins on the knockout mutant *lox1-2* and the complemented *lox1-2 35S:LOX1* lines. (A) Stomatal aperture measurements were determined on 2 h light-preincubated epidermal peels of 4–5-wk-old plants after 2.5 h incubation with 9- or 13-KODE (1 nM). Data represent means ± SD obtained of three independent experiments. (B) *Pst* DC3000 growth measurements were performed at day 3 on 4-wk-old plants either infiltrated with Mock or 9-KODE (150 µM) 3 h prior to spray inoculation with the bacterial suspension (5.10^7^ cfu/mL). Data are means ± SD of two independent experiments.
(TIF)

Figure S8 Stomatal closure responses to ABA. Dose response effects of ABA on 4–5-wk-old plants of Arabidopsis (Col-0). Measurements were performed on 2 h light-preincubated epidermal peels after 2.5 h incubation. Data represent means ± SD of three independent experiments.
(TIF)

Figure S9 Stomatal closure responses of the *slac1-3* mutant line to ABA, flg22, and 13-KODE. Stomatal aperture measurements were performed on 2 h light pre-incubated epidermal peels of 4–5-wk-old WT (Col-0) and *slac1-3* mutant plants after 2.5 h incubations with ABA (1 µM), flg22 (5 µM), and 13-KODE (1 nM). Data represent means ± SD of three independent experiments. Values marked with asterisks were significantly different from those of the corresponding WT controls.
(TIF)

Methods S1 Characterization of the *lox6-1* mutant line, quantitative real-time PCR, and bacterial growth in plants pretreated with 9-KODE.
(DOC)

Table S1 Sets of primers used to examine *LOX* gene expressions by RT-PCR.
(DOC)

Table S2 Oxylipins and other chemicals used in this study.
(DOC)

## Abbreviations

COI1: Coronatine Insensitive 1
FAH: fatty acid hydroperoxide
flg22: flagellin 22
JA-Ile: Jasmonate isoleucine
LOX: lipoxygenase
MAPK: mitogen-activated protein kinase
MS: Murashige & Skoog
OST1: Open Stomata 1
PAMP: pathogen-associated molecular pattern
*Pst*: *Pseudomonas syringae pv tomato*
PUFA: polyunsaturated fatty acid
RES: reactive electrophile species
ROS: reactive oxygen species
SD: standard deviation (a list of the abbreviations of all compounds used in this study is given in Table S2)
