# Supplementary figures and images for: An Abscisic Acid-Independent Oxylipin Pathway Controls Stomatal Closure and Immune Defense in Arabidopsis
Source: PLoS Biol. 2013 Mar 19;11(3):e1001513. doi: 10.1371/journal.pbio.1001513 (PMC3602010; doi:10.1371/journal.pbio.1001513)

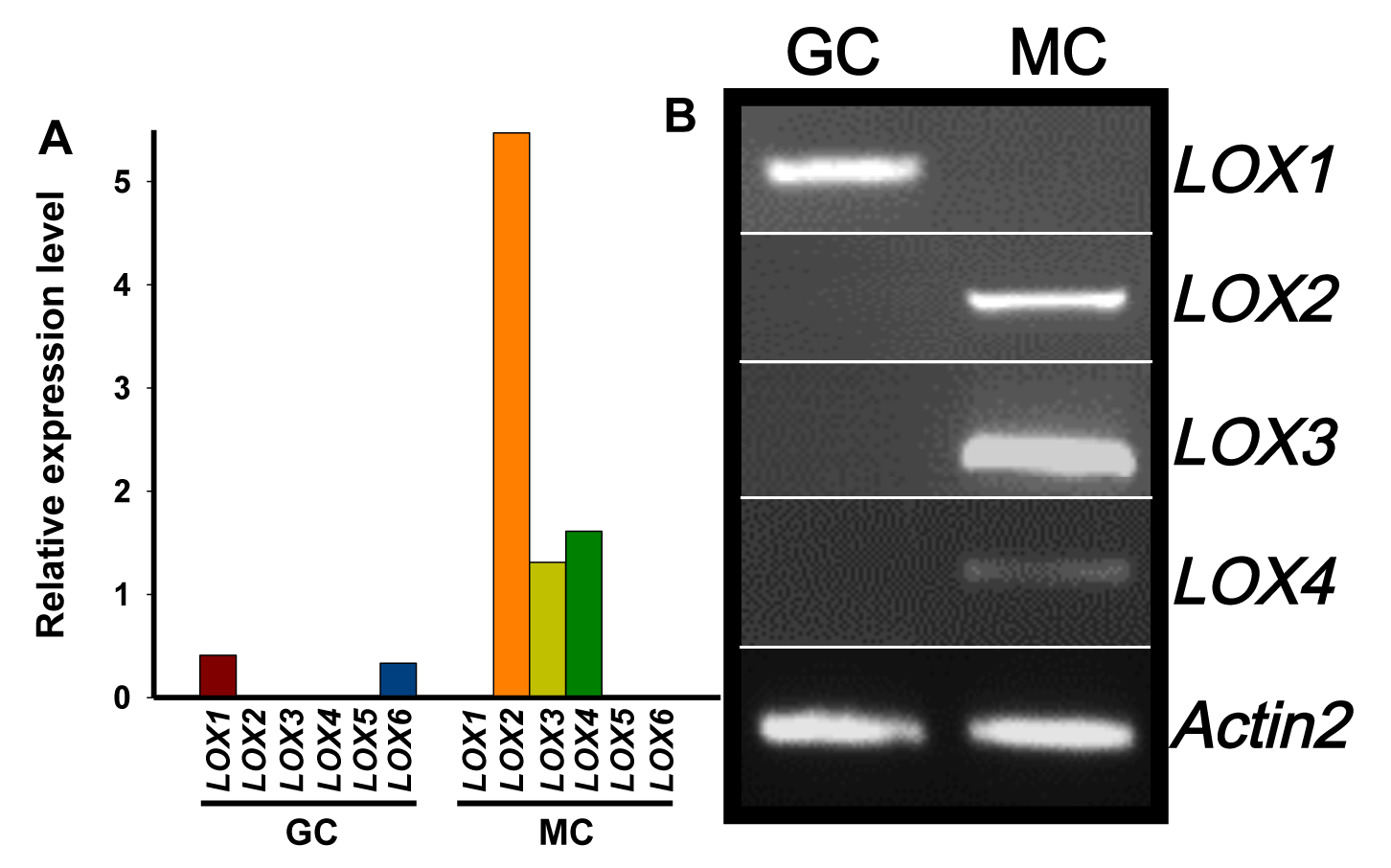

Supplement: Figure S1 — LOX gene expression analyses in leaf cells of Arabidopsis. (A) Data were extracted from microarray expression analyses published by Leonhardt et al. [30]. Expression levels of each gene in guard cells (GC) and mesophyll cells (MC) were normalized to 5S rRNA subunit. (B) RT-PCR of LOX expressed in leaves with RNA extracted from highly purified guard cell (GC) and mesophyll cell (MC) protoplasts. The Actin2 gene was used as a control. (TIF) [file pbio.1001513.s001.tif]

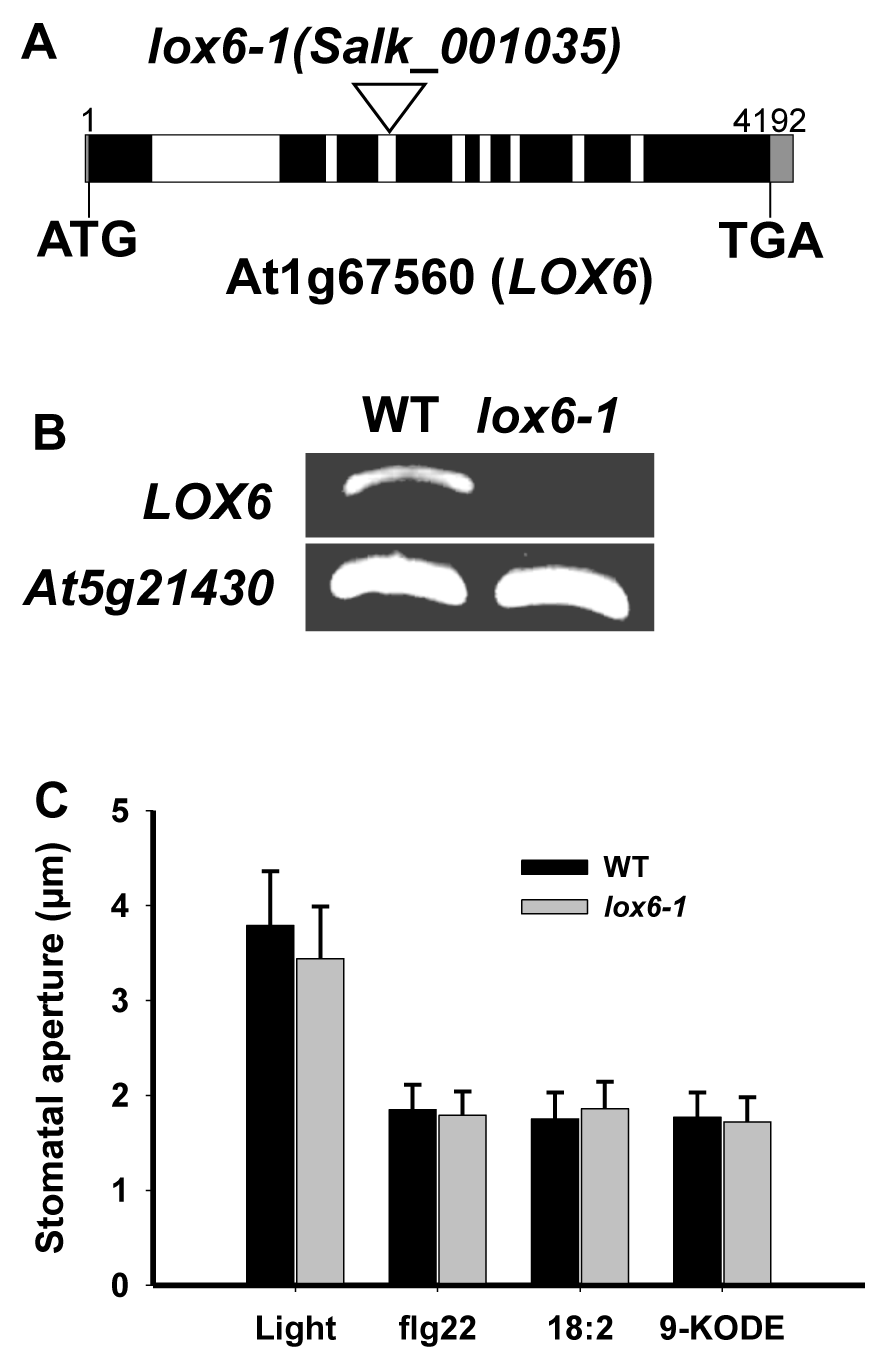

Supplement: Figure S2 — Stomatal closure responses of the lox6-1 mutant line to flg22, fatty acids, and 9-KODE. (A) Scheme of LOX6 genomic locus with exons represented as black boxes. The position of the T-DNA insertion is indicated. (B) RT-PCR of RNA isolated from leaves. Gene At5g21430 encoding the subunit U of NADH dehydrogenase-like complex (NDH-U) was used to normalize transcript levels in each sample. Gene-specific primer sets used for the evaluation of RNA are shown in Table S1. (C) Stomatal aperture measurements were performed on 2 h light-preincubated epidermal peels of 4–5-wk-old WT (Col-0) and lox6-1 mutant plants after 2.5 h incubation with flg22 (5 µM) linoleic acid (18∶2, 100 nM) and 9-KODE (1 nM). (TIF) [file pbio.1001513.s002.tif]

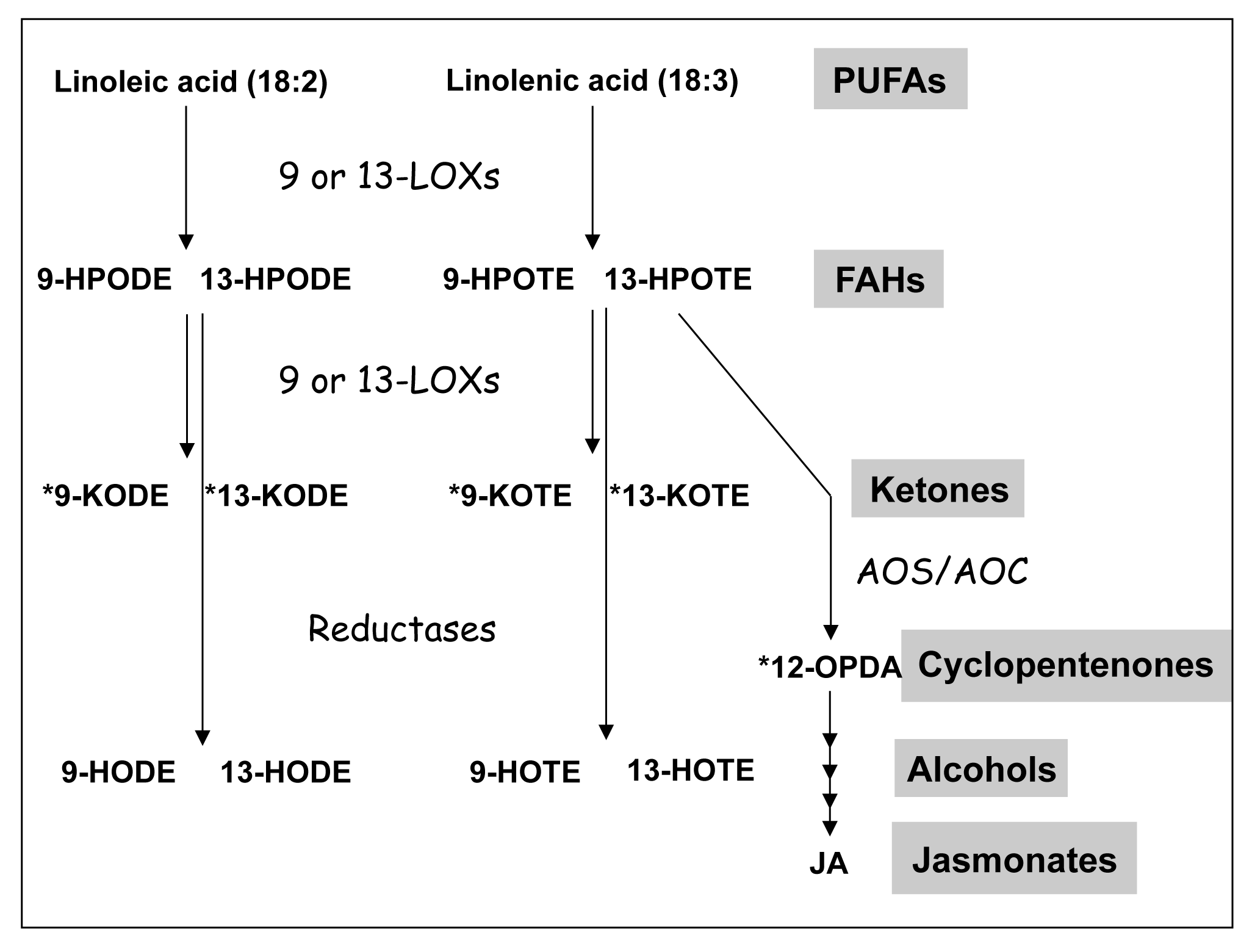

Supplement: Figure S3 — Simplified oxylipin pathway. Only oxylipins are described whose activity has been assessed on stomata of Arabidopsis. Fatty acid hydroperoxides (FAHs: 9/13-HPOD(T)E) and ketones (9/13-KOD(T)E) are synthesized in Arabidopsis by 9-specific (LOX1 and LOX5) and 13-specific LOXs (LOX2, LOX3, LOX4, and LOX6), whereas the alcohols (9/13-HOD(T)E) result from reduction of FAHs by reductases. Metabolization of 13-HPOTE by allene oxyde synthase (AOS) and allene oxyde cyclase (AOC) leads to the formation of the cyclopentenone (12-OPDA) and jasmonates (JAs). RES oxylipins are marked with an asterisk. Full names of these metabolites are given in Table S2. (TIF) [file pbio.1001513.s003.tif]

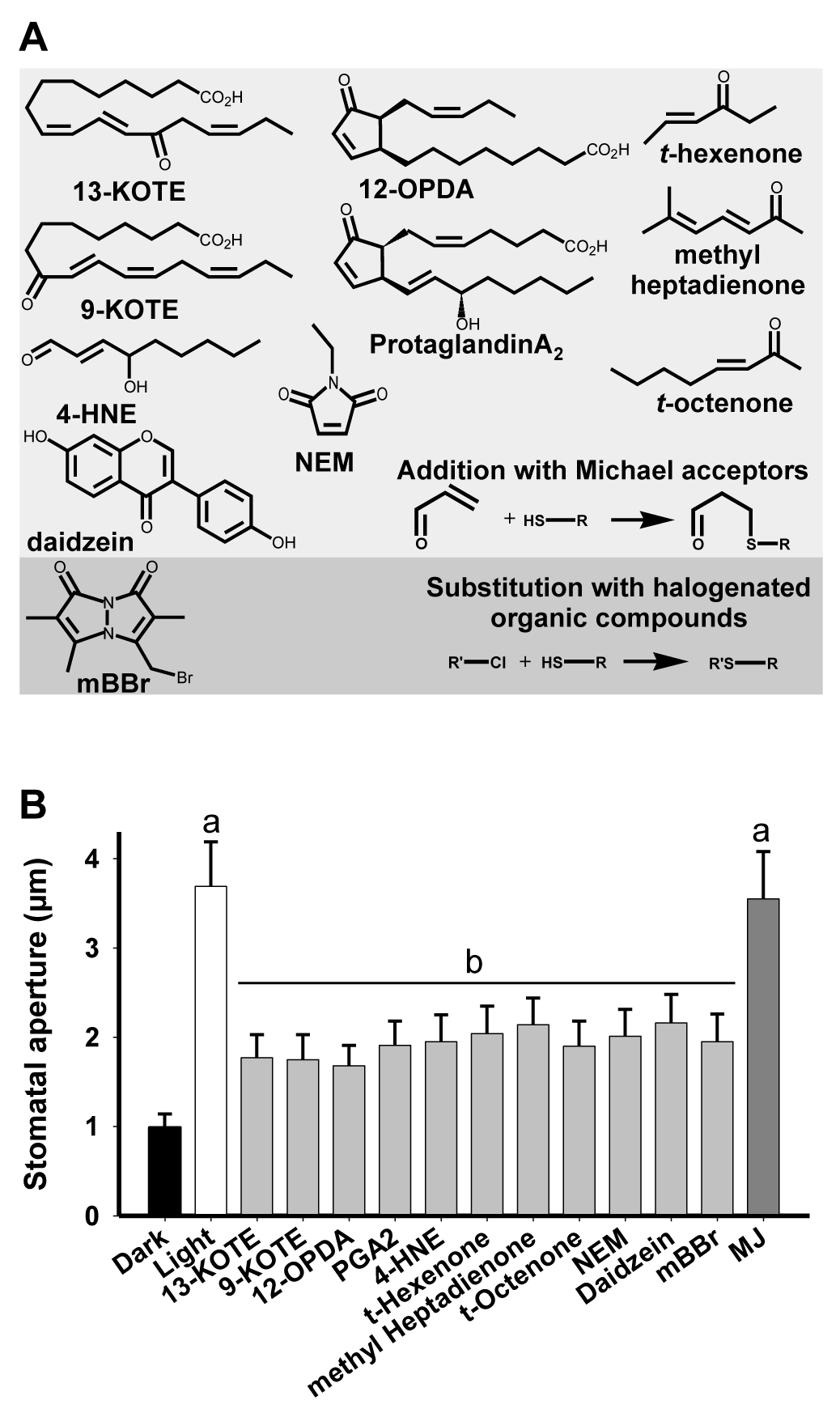

Supplement: Figure S4 — Stomatal closure responses to thiol-reagents and MJ. (A) Structures of natural and synthetic compounds inducing stomatal closure when incubated for 2.5 h on epidermal peels from 4–5-wk-old Col-0 plants. (B) Raw data showing the effect of compounds tested at a concentration of 1 nM (gray bars). For comparison, MJ has been tested at 100 µM (dark gray bar). Data represent means ± SD of three independent experiments, and values marked with identical letters were not statistically different. (TIF) [file pbio.1001513.s004.tif]

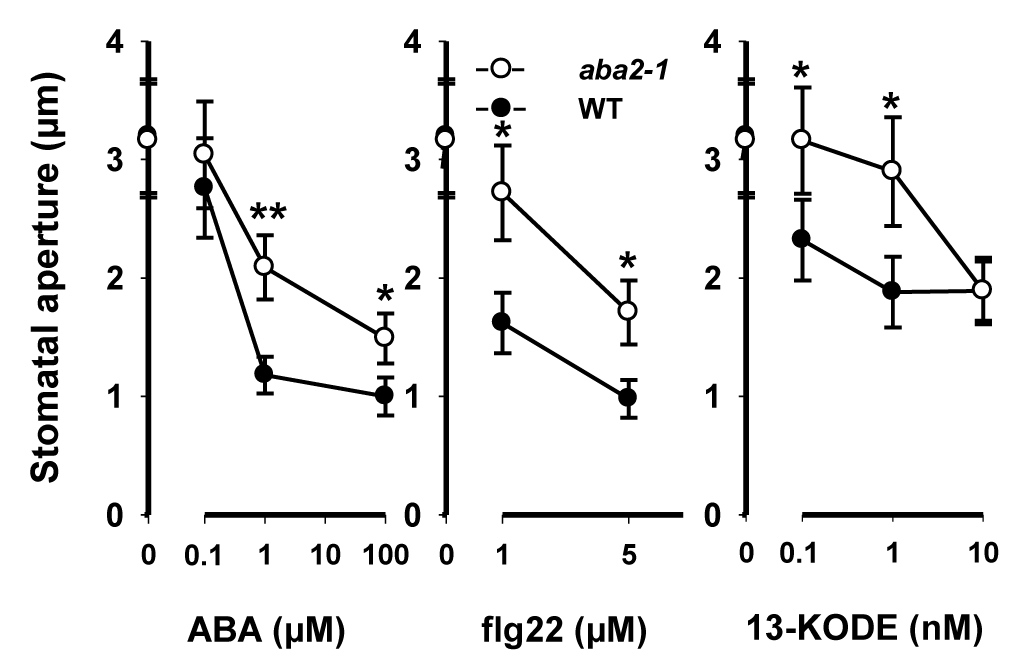

Supplement: Figure S5 — Comparative effects of ABA, flg22, and 13-KODE on stomata of the Arabidopsis aba2-1 mutant line defective in ABA synthesis. Dose response effects of ABA, flg22, and 13-KODE were performed on 2 h light-preincubated epidermal peels of 4–5-wk-old mutant plants, abi1-1 and their corresponding WT ecotype Col-0, after 2.5 h incubation with compounds. Data represent means ± SD of three independent experiments. (TIF) [file pbio.1001513.s005.tif]

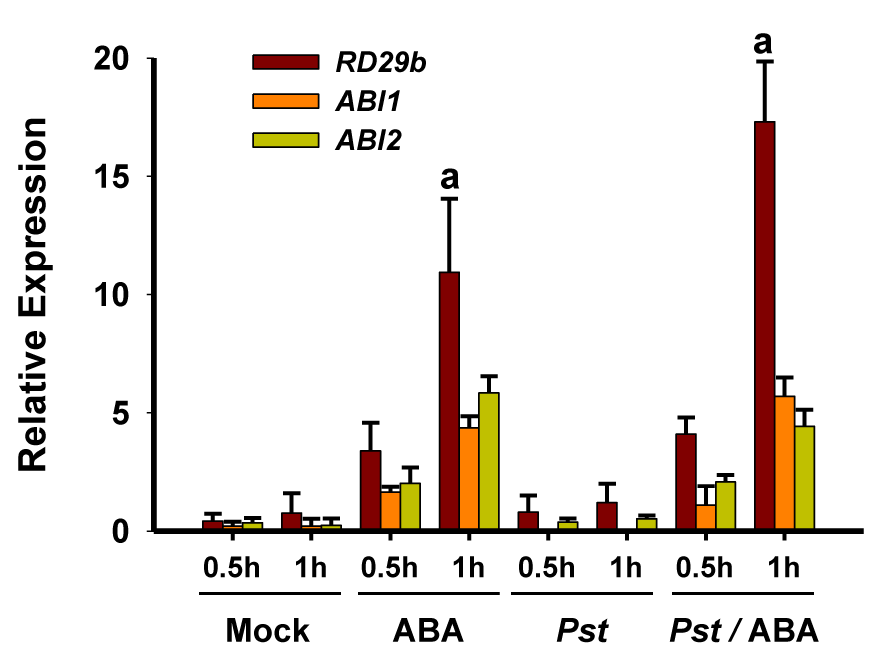

Supplement: Figure S6 — Early gene expression in response to ABA and Pst DC3000. Three-week-old plants Col-0 were sprayed with ABA (100 µM), Pst DC3000 suspensions (109 cfu/mL), Pst DC3000 followed by ABA 30 min later or Mock (0.1%, v/v ethanol in water). After treatments, plants were sampled at 0.5 and 1 h for cDNA synthesis and quantitative qRT-PCR. The transcript levels of ABA-responsive genes, RD29b (At5g52300), ABI1 (At4g26080), and ABI2 (At5g57050), were normalized using EF1 (At5g60390), and data represent means ± SD of three independent experiments. Values marked with identical letters were not statistically different. (TIF) [file pbio.1001513.s006.tif]

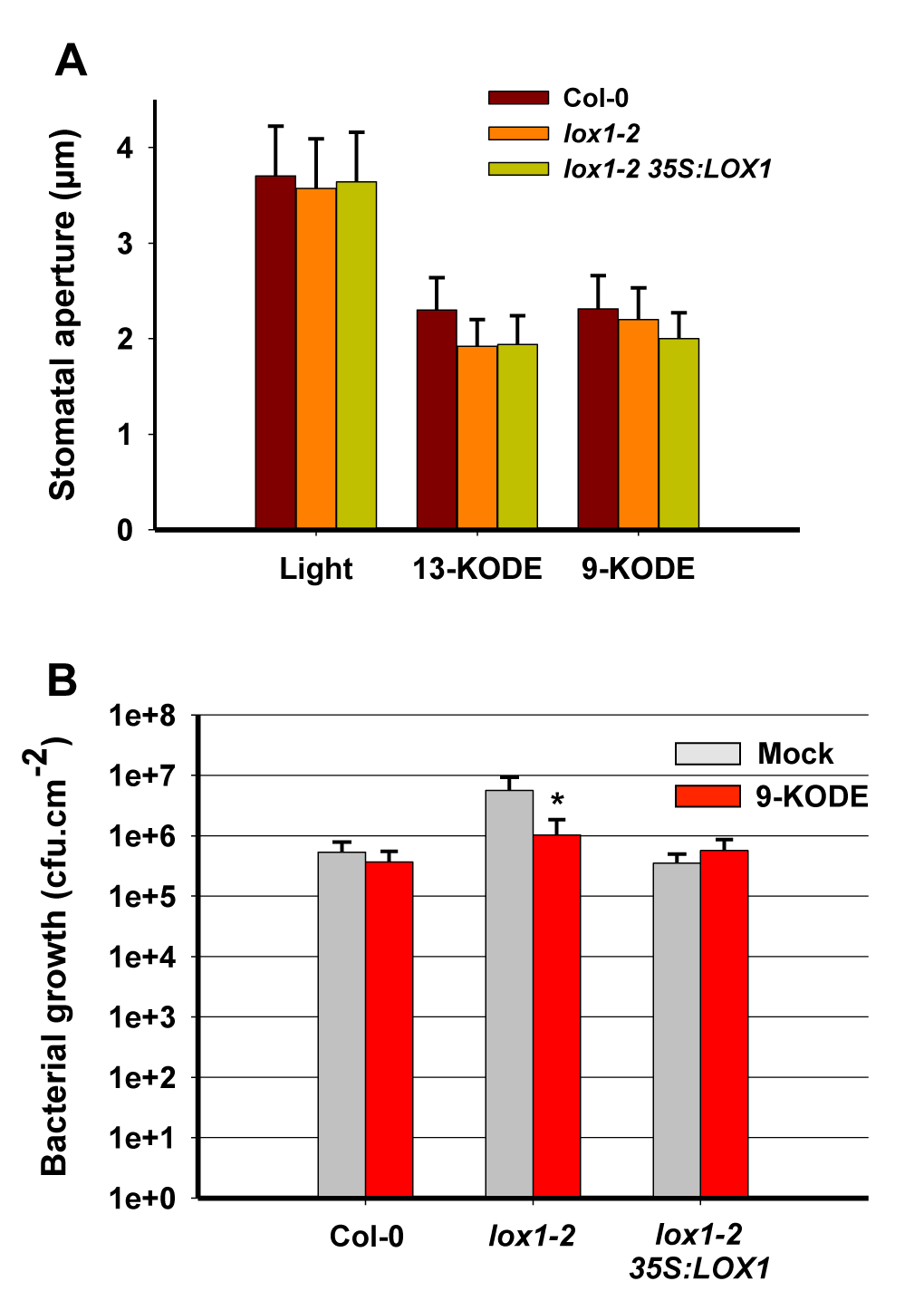

Supplement: Figure S7 — Effects of RES oxylipins on the knockout mutant lox1-2 and the complemented lox1-2 35S:LOX1 lines. (A) Stomatal aperture measurements were determined on 2 h light-preincubated epidermal peels of 4–5-wk-old plants after 2.5 h incubation with 9- or 13-KODE (1 nM). Data represent means ± SD obtained of three independent experiments. (B) Pst DC3000 growth measurements were performed at day 3 on 4-wk-old plants either infiltrated with Mock or 9-KODE (150 µM) 3 h prior to spray inoculation with the bacterial suspension (5.107 cfu/mL). Data are means ± SD of two independent experiments. (TIF) [file pbio.1001513.s007.tif]

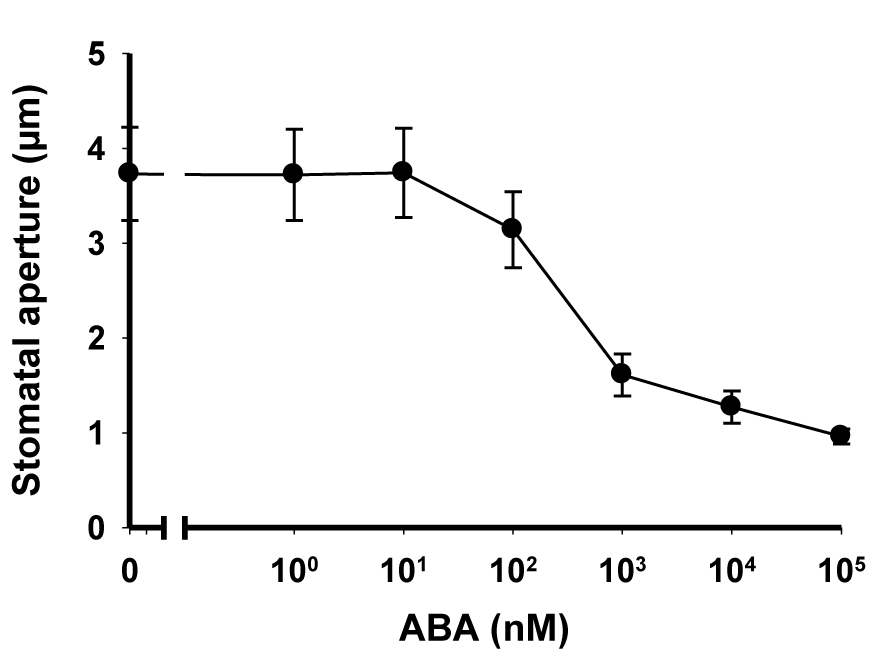

Supplement: Figure S8 — Stomatal closure responses to ABA. Dose response effects of ABA on 4–5-wk-old plants of Arabidopsis (Col-0). Measurements were performed on 2 h light-preincubated epidermal peels after 2.5 h incubation. Data represent means ± SD of three independent experiments. (TIF) [file pbio.1001513.s008.tif]

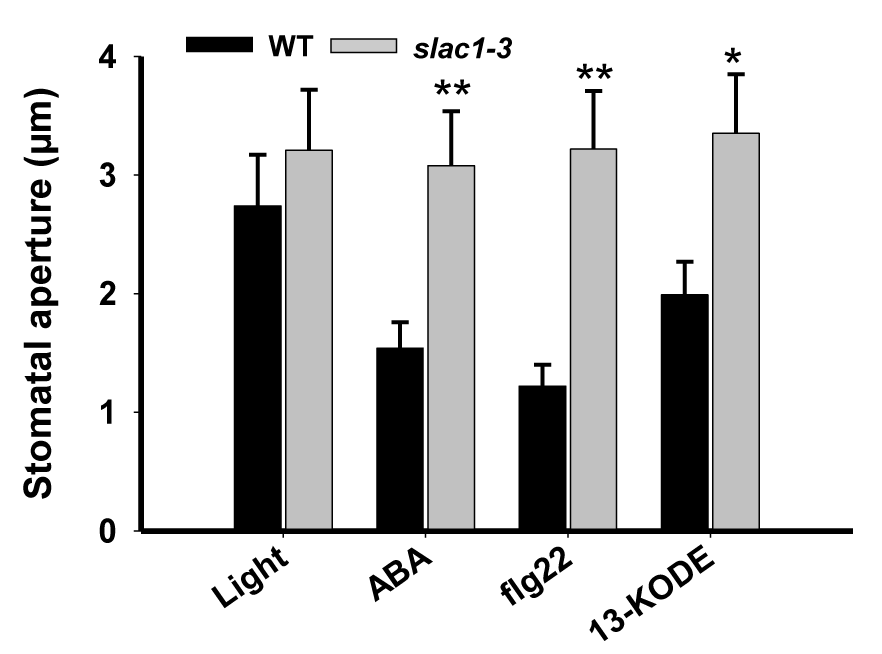

Supplement: Figure S9 — Stomatal closure responses of the slac1-3 mutant line to ABA, flg22, and 13-KODE. Stomatal aperture measurements were performed on 2 h light pre-incubated epidermal peels of 4–5-wk-old WT (Col-0) and slac1-3 mutant plants after 2.5 h incubations with ABA (1 µM), flg22 (5 µM), and 13-KODE (1 nM). Data represent means ± SD of three independent experiments. Values marked with asterisks were significantly different from those of the corresponding WT controls. (TIF) [file pbio.1001513.s009.tif]
