# Supplementary material for: An Abscisic Acid-Independent Oxylipin Pathway Controls Stomatal Closure and Immune Defense in Arabidopsis
Source: PLoS Biol. 2013 Mar 19;11(3):e1001513. doi: 10.1371/journal.pbio.1001513 (PMC3602010; doi:10.1371/journal.pbio.1001513)
Supplement: Methods S1 — Characterization of the lox6-1 mutant line, quantitative real-time PCR, and bacterial growth in plants pretreated with 9-KODE. (DOC) [file pbio.1001513.s010.doc]

Supporting methods

Characterization of the *lox6-1* mutant line

The SALK line SALK_001035 (*lox6-1*) was distributed by the Nottingham Arabidopsis Stock Centre ([http://arabidopsis.info](http://arabidopsis.info/)). Homozygous insertion mutants were identified by PCR using T-DNA and gene-specific primer sets as described on the T-DNA Express homepage. To evaluate whether the isolated mutants were mRNA null mutants, semi-quantitative RT-PCR was performed using gene-specific primers (sequences of primer sets used are shown in Supplemental Table S1).

Quantitative Real-Time-PCR

Arabidopsis seedlings were grown for 3 weeks at 22°C under a 16 h-light/8 h-dark photoperiod with a light intensity of 150 µE.m^-2^ .s^-^1 in vertically oriented square Petri dishes (120 mm x 120 mm, Greiner) containing 0.5x MS medium, pH 5.7, 1% (w/v) sucrose and 1.5% (w/v) BactoAgar (Gifco). After spray treatments with 100 µM ABA, bacterial suspension (10^9^ cfu/mL) or bacterial suspension followed 30 min later by ABA and incubation at 22°C under light, seedlings were collected at different time points for RNA extraction, which was performed using Trizol reagent according to manufacturer’s instructions (Invitrogen). The RNA was then subjected to treatment with TURBO DNase (Ambion, Applied Biosystems). cDNA synthesis was done using the SuperScript III first strand synthesis system for RT-PCR (Invitrogen). Quantitative RT-PCR was performed in 384-well plates using the Light Cycler480 SYBR Green I Master and the LightCycler 480 real-time PCR system (Roche Diagnostics). The accumulation of each transcript was measured in three independent biological samples with three technical replicates. *EF1* expression was used to normalize the transcript levels. Primers used were as follows: for *RD29b* (At5g52300), forward, 5’-CACCACCGTTGGGACTATG-3’ and reverse, 5’-GCAAAACCCCAAATCTTCAG-3’; for *ABI1* (At4g26080), forward, 5’-GAGGCAGAGAGGGTCCTTTT-3’ and reverse, 5’-AGCCCGGGAAGAAAAATACA-3’; for *ABI2* (At5g57050), forward, 5’-AAGATCCTGCAGCAATGTCC-3’ and reverse, 5’-TCCCTTCAAATCAACCACTACC-3’; for *EF1* (At5g60390), forward, 5’-TGAGCACGCTCTTCTTGCTTTCA-3’ and reverse 5’-GGTGGTGGCATCCATCTTGTTACA-3’. Bars represent mean values from three independent experiments.

Bacterial growth in plants pretreated with 9-KODE

Plants were grown on soil in a controlled environment chamber under an 8 h light regime (150–200 µE.m^-2^ .s^-1^) at 22°C and 65% relative humidity. *Pst* DC3000 was grown for 24 h at 28°C on NYGA solid medium supplemented with 50 µg/mL Rifampicin. For examination of the bacterial growth after ketone treatment three leaves of each independent 4-week-old plant were injected with 150 µM 9-KODE in 10 mM MgCl_2_ with 0.03% (v/v) Silwet L-77 (Lehle Seeds) and 0.1% (v/v) ethanol 3 h prior to spray-inoculation with *Pseudomonas syringae* DC3000 suspensions at 5.10^7^ cfu/mL in 10 mM MgCl_2_ with 0.03% (v/v) Silwet L-77 and 0.1% ethanol. *In planta* bacterial titers were determined at day 3 by shaking leaf discs in 10 mM MgCl_2_ with 0,01% (v/v) Silwet L-77 at 28°C for 1 h as previously described [70]. At least six plants per genotype were used for each sampling and the experiment was performed in duplicate.
