# Supplementary material for: An Abscisic Acid-Independent Oxylipin Pathway Controls Stomatal Closure and Immune Defense in Arabidopsis
Source: PLoS Biol. 2013 Mar 19;11(3):e1001513. doi: 10.1371/journal.pbio.1001513 (PMC3602010; doi:10.1371/journal.pbio.1001513)
Supplement: Table S1 — Sets of primers used to examine LOX gene expressions by RT-PCR. (DOC) [file pbio.1001513.s011.doc]

**Table S1.** **Sets of primers used to examine *LOX gene* expressions by RT-PCR.**

| Locus and gene name | Upper primer sequence | Lower primer sequence | Amplicon length (bp) |
| --- | --- | --- | --- |
| At1g55020 *LOX1* | 5’ AGCCGTCAGTACATGC 3’ | 5’ AGACCTCGTCGCTAGA 3’ | 151 |
| At3g45140 *LOX2* | 5’ GTCTATGGTAAACTATGGAGG 3’ | 5’ TCATCTGTCGGATCTTCCGTT 3’ | 404 |
| At1g17420 *LOX3* | 5’ CCATGGTCCAAAACATC 3’ | 5’ GGGAGATGAAACTCGCGT 3’ | 821 |
| At1g72520 *LOX4* | 5’ TCACGAGTTTTATCGAAG 3’ | 5’ ACGGCTAGGATCACGA 3’ | 251 |
| At3g18780 *Actin2* | 5’ CGCTATGTATGTCGCCA 3’ | 5’ CAATTTCCCGCTCTGC 3’ | 236 |
| At1g67560 *LOX6* | 5’ CAGTTCTAGGTGTTCCAGAGA 3’ | 5’ AAGGTATCTCTCTTGATTTCCTC 3’ | 155 |
| At5g21430 *NADH dehydrogenase* | 5’ GAGTCATATACGATATTGTC 3’ | 5’ TCCTCGTTGGTCCTACATCTTC 3’ | 170 |
