# Supplementary material for: An Abscisic Acid-Independent Oxylipin Pathway Controls Stomatal Closure and Immune Defense in Arabidopsis
Source: PLoS Biol. 2013 Mar 19;11(3):e1001513. doi: 10.1371/journal.pbio.1001513 (PMC3602010; doi:10.1371/journal.pbio.1001513)
Supplement: Table S2 — Oxylipins and other chemicals used in this study. (DOC) [file pbio.1001513.s012.doc]

**Table S2**. **Oxylipins and other chemicals used in this study.**

| Compound name | Purity (%) | Supplier |
| --- | --- | --- |
| 9-HPODE, (9*S*,10*E*,12*Z*)-9-hydroperoxy-10,12-octadecadienoic acid |  |  |
| 9-KODE, (10*E*,12*Z*)-9-oxo-10,12-octadecadienoic acid |  |  |
| 13-HPODE, (9*Z*,11*E*,13*S*)-13-hydroperoxy-9,11-octadecadienoic acid |  |  |
| 13-KODE, (9*Z*,11*E*)-13-oxo-9,11-octadecadienoic acid. | > 97 | Larodan Fine Chemical |
| 9-KOTE, (10*E*,12*Z*,15*Z*)-9-oxo-10,12,15-octadecatrienoic acid |  |  |
| 13-KOTE, (9*Z*,11*E*,15*Z*)-13-oxo-9,11,15-octadecatrienoic acid; |  |  |
| 12-OPDA, 12-oxo-10,15(*Z*)-phytodienoic acid |  |  |
| 4-HNE, (±)-4-hydroxy-2*E*-nonenal | > 98 | Cayman Chemical |
| methyl heptadienone, 6-methyl-3,5-heptadien-2-one | 97 | Alfa Aesar |
| *t*-octenone, 3-octen-2-one |  |  |
| t-hexenone, 4-hexen-3-one | > 90 |  |
| daidzein | 98 |  |
| NEM, *N*-ethylmaleimide | ≥ 98 |  |
| PCMB, 4-chloromercuribenzoic acid | ≥ 95 |  |
| mBBr, Monobromobimane | ≥ 95 |  |
| L-sulforaphane | ≥ 95 |  |
| ABA, (±) abscisic acid | 98.5 |  |
| NAC, *N*-Acetyl-L-cysteine | ≥ 99 | Sigma-Aldrich |
| COR, **Cyclopropanecarboxylic acid,2-ethyl-1-[[(6-ethyl-2,3,3a,6,7a-hexahydro-1-oxo-1H-inden-4-yl)carbonyl] amino]** | > 95 |  |
| linoleic acid, 18:2, (9*Z*,12*Z*)-octadecadienoic acid | ≥ 99 |  |
| linolenic acid, 18:3, (6*Z*,9*Z*,12*Z*)-octadecatrienoic acid | ≥ 99 |  |
| oleic acid, 18:1, (9Z)-octadecenoic acid | ≥ 99 |  |
| MJ, Methyl Jasmonate, **3-Oxo-2-(2-pentenyl)cyclopentaneacetic acid, methyl ester** | 95 |  |
| ProstaglandinA2, (5*Z*,13*E*, 15*S*)-15-hydroxy-9-oxoprosta-5,10,13-trien-1-oic acid | 95 |  |
| SA, Salycilic acid | ≥ 99 |  |
| tBuOOH, *Tert*-butyl hydroperoxide, 70% in water |  |  |
| H_2_O_2_, Hydrogen peroxide, 30% in water | ____ |  |
